# Targeting inhibition of prognosis-related lipid metabolism genes including CYP19A1 enhances immunotherapeutic response in colon cancer

**DOI:** 10.1186/s13046-023-02647-8

**Published:** 2023-04-13

**Authors:** Lilong Liu, Min Mo, Xuehan Chen, Dongchen Chao, Yufan Zhang, Xuewei Chen, Yang Wang, Nan Zhang, Nan He, Xi Yuan, Honglei Chen, Jing Yang

**Affiliations:** 1 Department of Pharmacology and Hubei Province Key Laboratory of Allergy and Immune-related Diseases, School of Basic Medical Sciences, Wuhan University, 185 Donghu Road, Wuhan, 430071 China; 2 Department of Laboratory Medicine, Zhongnan Hospital of Wuhan University, Wuhan, 430071 China; 3 Department of Pathology, School of Basic Medical Sciences, Wuhan University, Wuhan, 430071 China

**Keywords:** Lipid metabolism, CYP19A1, Tumor immune microenvironment, Immunotherapeutic response, Colon cancer

## Abstract

**Background:**

Lipid metabolic reprogramming in colon cancer shows a potential impact on tumor immune microenvironment and is associated with response to immunotherapy. Therefore, this study aimed to develop a lipid metabolism-related prognostic risk score (LMrisk) to provide new biomarkers and combination therapy strategies for colon cancer immunotherapy.

**Methods:**

Differentially expressed lipid metabolism-related genes (LMGs) including cytochrome P450 (CYP) 19A1 were screened to construct LMrisk in TCGA colon cancer cohort. The LMrisk was then validated in three GEO datasets. The differences of immune cell infiltration and immunotherapy response between LMrisk subgroups were investigated via bioinformatic analysis. These results were comfirmed by in vitro coculture of colon cancer cells with peripheral blood mononuclear cells, human colon cancer tissue microarray analysis, multiplex immunofluorescence staining and mouse xenograft models of colon cancer.

**Results:**

Six LMGs including CYP19A1, ALOXE3, FABP4, LRP2, SLCO1A2 and PPARGC1A were selected to establish the LMrisk. The LMrisk was positively correlated with the abundance of macrophages, carcinoma-associated fibroblasts (CAFs), endothelial cells and the levels of biomarkers for immunotherapeutic response including programmed cell death ligand 1 (PD-L1) expression, tumor mutation burden and microsatellite instability, but negatively correlated with CD8^+^ T cell infiltration levels. CYP19A1 protein expression was an independent prognostic factor, and positively correlated with PD-L1 expression in human colon cancer tissues. Multiplex immunofluorescence analyses revealed that CYP19A1 protein expression was negatively correlated with CD8^+^ T cell infiltration, but positively correlated with the levels of tumor-associated macrophages, CAFs and endothelial cells. Importantly, CYP19A1 inhibition downregulated PD-L1, IL-6 and TGF-β levels through GPR30-AKT signaling, thereby enhancing CD8^+^ T cell-mediated antitumor immune response in vitro co-culture studies. CYP19A1 inhibition by letrozole or siRNA strengthened the anti-tumor immune response of CD8^+^ T cells, induced normalization of tumor blood vessels, and enhanced the efficacy of anti-PD-1 therapy in orthotopic and subcutaneous mouse colon cancer models.

**Conclusion:**

A risk model based on lipid metabolism-related genes may predict prognosis and immunotherapeutic response in colon cancer. CYP19A1-catalyzed estrogen biosynthesis promotes vascular abnormality and inhibits CD8^+^ T cell function through the upregulation of PD-L1, IL-6 and TGF-β *via* GPR30-AKT signaling. CYP19A1 inhibition combined with PD-1 blockade represents a promising therapeutic strategy for colon cancer immunotherapy.

**Supplementary Information:**

The online version contains supplementary material available at 10.1186/s13046-023-02647-8.

## Introduction

Colon cancer is one of the most common human malignancies in the world [1]. Despite the fact that clinical treatment for colon cancer has been improved with the development of immune checkpoint inhibitors (ICIs), the majority of colon cancer patients have limited response to ICI therapies [2]. Emerging biomarkers such as tumor mutational burden (TMB), inflammatory tumor microenvironment (TME) and microsatellite instability (MSI) have been identified to predict therapeutic benefit in colon cancer [3]. Unfortunately, drawbacks still remained in current biomarkers [3]. Therefore, it is important to excavate the predictive biomarkers for immunotherapy response and find a novel strategy for sensitizing ICIs in colon cancer.

Lipid metabolic reprogramming promotes tumor growth, angiogenesis and metastasis [4]. A prognostic signature of nine lipid metabolism-related genes (LMGs), including CDIPT, MTMR7, PIK3CB, PIK3C2G, ARSE, ARSJ, GLA, GLB1 and UGCG, has been established in diffuse gliomas [5]. The model including the four LMGs (ABCA1, ACSL1, AGPAT1 and SCD) is proposed as a prognostic marker of colon cancer with stage II [6], but not effective in all stages of colon cancer [7]. A very recent study has shown an 8-gene prognostic signature based on LMGs in colon adenocarcinoma [8]. However, the prognostic prediction model of multiple LMGs in colon cancer has only begun to be appreciated.

Metabolic reprogramming of tumor cells induces metabolic stress in tumor-infiltrating immune cells and stromal cells, and thereby impairs antitumor immune responses [9]. Targeted reprogramming of lipid metabolism inhibits tumor cell growth, alleviates the immunosuppressive TME and improves response to ICI therapy [10]. A study has shown that cyclooxygenase (COX) enzyme inhibitor aspirin in combination with anti-programmed cell death protein 1 (PD-1) treatment exhibits a synergistic effect in reducing tumor growth [11]. Our recent study has also shown that inhibition of COX-2 catalyzed metabolism of arachidonic acid (AA) by melafolone promotes anti-PD-1 therapy in lung cancer through PD-L1 downregulation [12]. Avasimibe, an inhibitor of cholesterol esterification enzyme, significantly empowers the anti-tumor response of CD8^+^ T cells, and its combination with anti-PD-1 antibody has a better efficacy in melanoma [13]. Some clinical studies have further confirmed that targeted metabolic reprogramming enhances the anti-tumor efficacy of ICIs [14]. Therefore, it is essential to investigate the new strategy of targeting tumor lipid metabolism to alleviate the immunosuppressive TME and to enhance anti-tumor immunotherapy.

Cytochrome P450 (CYP) 19A1 encodes aromatase, an isoenzyme of estrogen biosynthesis, and is overexpressed in colon cancer tissues [15]. Aromatase inhibitors, including letrozole, anastrozole and exemestane, inhibit the synthesis of estrogen from androgen by binding to the aromatase, and thereby block tumor cell proliferation [15]. Aromatase inhibitors are widely used to treat post-menopausal hormone-sensitive breast cancer [16]. Exemestane exhibits great growth inhibitory potentials in gastric cancer when administered in combination therapy with 5-fluorouracil [17]. Letrozole plus mTOR inhibitor everolimus results in a better progression free survival in patients with breast cancer, accompanied by decreases in Ki-67 index and tumor-infiltrating regulatory T cells, and an increase in tumor-specific CD8^+^ T cells [18–20]. Recently, the combination of exemestane and CTLA-4 monoclonal antibody tremelimumab in breast cancer has entered a phase II clinical trial [21]. However, the effect of aromatase inhibitors on the immunotherapeutic response of colon cancer is not well understood.

In this study, we utilized The Cancer Genome Atlas (TCGA) cohort as a training set to develop a lipid metabolism-related gene prognostic risk score (LMrisk) in colon cancer, and the significant prognostic values of the model were then validated in three Gene Expression Omnibus (GEO) testing sets. The relationships of LMrisk with 36 immune cell signatures and predictive biomarkers for immunotherapeutic response were evaluated. In particular, the bioinformatic findings were confirmed using human colon cancer tissue samples, in vitro coculture of colon cancer cells with peripheral blood mononuclear cells (PBMCs), and mouse xenograft models of colon cancer. Our study develops a novel lipid metabolism-related risk model to predict immunotherapeutic response, and elucidates the molecular mechanism by which CYP19A1-catalyzed estrogen synthesis mediates immune escape, providing new targets and candidates for sensitizing colon cancer immunotherapy.

## Methods

### Data and resources

The RNA-sequencing data and clinical information of colon cancer patients were downloaded from the TCGA database (https://portal.gdc.cancer.gov). The RNA expression profiles contained 494 samples. We obtained 453 colon cancer samples and 41 adjacent normal tissue samples. 435 colon cancer patients with survival data were included in the following study. Gene expression profiles of GSE41258 dataset based on the Affymetrix human genome U133A array platform, GSE38832 and GSE39582 datasets based on the Affymetrix human genome U133 Plus 2.0 array platform and clinical data were downloaded from the GEO database (https://www.ncbi.nlm.nih.gov/geo/). A list of LMGs was collected from the “metabolism of lipids” in Reactome (https://reactome.org/download-data/). Genes not included in TCGA or GEO databases were excluded, and 731 genes related to lipid metabolism were obtained.

### Differential expression analysis of LMGs and functional annotation

The “edgeR” [22] package was used to identify differentially expressed LMGs between the tumor and adjacent normal tissue samples. Adjusted *P*-value < 0.05 and |log2 (fold change) |> 1 were chosen as the cut-off threshold. The protein–protein interaction network of differentially expressed LMGs was analyzed by STRING database (https://string-db.org/). Principal component analysis (PCA) was utilized to analyze the expression pattern in the colon cancer and normal tissues. Gene Ontology (GO) and Kyoto Encyclopedia of Genes and Genomes (KEGG) enrichment analysis were performed with the differentially expressed LMGs by using the “clusterProfiler” R package [23]. False discovery rate < 0.05 was considered statistically significant.

### Construction and verification of the LMrisk

Univariate Cox regression analysis was performed to identify the prognosis-related LMGs. The *P*-value < 0.05 in univariate Cox regression analysis was considered statistically significant. The “glmnet” R package was used to perform a least absolute shrinkage and selection operator (LASSO)-Cox regression model analysis. The weighted LASSO-Cox coefficients based on individual gene expression levels were used to calculate the lipid metabolism-related risk score (LMrisk) as follows: LMrisk = ∑ (expression of gene_*i*_ × Coefficient of gene_*i*_). The patients in the TCGA database were stratified into the low- and high-LMrisk groups according to median LMrisk value, and their survival were analyzed using the Kaplan–Meier method. Log-rank test was used to compare the survival curves of two or more groups. The specificity and sensitivity of the LMrisk in predicting 3-, 5- and 10-year survival were determined by receiver operating characteristic (ROC) analysis using the “survivalROC” R package, and the areas under curves (AUC) were calculated. We used a similar approach in the GSE41258, GSE38832 and GSE39582 datasets to verify the applicability of the LMrisk. To study whether the LMrisk is an independent predictor for overall survival of colon cancer patients, univariate and multivariate Cox regression analyses were conducted. The LMrisk, age, gender and TNM stage were used as covariates.

### Construction and verification of prognostic nomogram

After testing for collinearity, all independent prognostic parameters were included in the construction of a nomogram to predict 3- and 5-year overall survival of colon cancer patients. Age, TNM stage and LMrisk were used to construct the nomogram using the “rms” and “survival” packages in R. The discrimination power of the predictive model was evaluated by Harrell’s concordance index (C-index). We calculated the C-index with 95% confidence interval using the bootstrap approach with 1000 resamples. Then, calibration curves were drawn to assess the consistency between actual and predicted survival. The nomogram performance was validated by ROC curves at 3 and 5 year using the TNM stage as control.

### Analysis of tumor immune signatures and function enrichment for LMrisk

The Estimation of Stromal and Immune cells in Malignant Tumors using Expression data (ESTIMATE) was used to evaluate the immune score, stromal score and ESTIMATE score of each sample [24]. Based on the gene expression data in the cancer tissues, the xCell and TIMER algorithms were applied to estimate the infiltration of immune cells in each sample [25]. The R package “maftools” was used to evaluate and sum the mutation data.

### Chemicals and reagents

Letrozole (T1590) was purchased from TargetMol (Bellingham, WA, USA). CYP19A1 siRNA (sc-41498) and CYP19A1 antibody (sc-374176) were purchased from Santa Cruz Biotechnology (Santa Cruz, CA, USA). PD-L1 (13684) antibody was purchased from Cell Signaling Technology (Danvers, MA, USA). PE-conjugated anti-CD3 antibody (555340), APC-conjugated anti-CD8 antibody (566852), PE-Cy7-conjugated anti-IFN-γ antibody (557643) were purchased from BD Biosciences (San Jose, CA, USA). FITC-conjugated anti-CD107a antibody (328606) and Zombie NIR (423105) were purchased from BioLegend (San Diego, CA, USA). Opal Polaris 7 Color IHC Detection Kit (NEL861001KT) was purchased from Akoya Bioscience (Menlo Park, CA, USA). Chitosan (CS, deacetylation 98%, Mw = 50 KDa) and sodium tripolyphosphate (TPP) were purchased from Sigma Chemical Co (St. Louis, MO, USA). Hyaluronic acid (HA) was purchased from Meryer Chemical Technology Co Ltd (Shanghai, China).

### Human tissue microarray analyses for CYP19A1 and PD-L1 expression

Human colon carcinoma tissue microarray (HCol-Ade180Sur-05) containing 90 patients with colon cancer and adjacent normal tissues were obtained from Shanghai Outdo Biotech Co., Ltd. Each sample dot with a diameter of 1.5 mm and a thickness of 4 μm was prepared according to a standard method. Immunohistochemistry (IHC) of human colon cancer tissue microarrays were performed by using the CYP19A1 (1:100) and PD-L1 (1:300) antibodies. Immunostaining was graded using a two-score system based on intensity score and proportion score as previously described [26]. The two scores were then multiplied to yield a total immunoreactivity score regarding the protein expression in a sample. IHC score was assessed independently by two pathologists, and a consensus of grading was reached. Patients were divided into high- and low-expression groups according to the median IHC score.

### Multiplex immunofluorescence analysis

Tumor tissues from 20 patients with colon cancer were obtained from Zhongnan Hospital, Wuhan University (Hubei, China) between 2020 and 2021. The histological diagnosis for each sample was reconfirmed using microscopic examination of hematoxylin/eosin-stained sections. All samples were collected from patients with informed consent, and all procedures were conducted with the approval of the Ethical Committee of the Medical School of Wuhan University and performed in accordance with relevant regulations and guidelines.

Quantitative multiplex immunofluorescence (mIF) was performed to characterize the immune landscape in human colon cancer tissues using Opal Polaris 7 Color IHC Detection Kits (NEL861001KT, Akoya Bioscience, CA, USA) as previously described [27]. Briefly, formalin-fixed paraffin-embedded sections were deparaffinized, followed by antigen retrieval with citrate acid buffer (pH 6.0)/Tris–EDTA buffer (pH 9.0) and then blocking with blocking/Ab diluent. Next, slides were incubated with primary antibodies against CYP19A1 (1:200), CD68 (Abcam, ab955, 1:800), CD31 (Abcam, ab24590, 1:300), α-SMA (Abcam, ab7817, 1:300), CD8 (DAKO, IR623, 1:5) and CK (AE1/AE3) (DAKO, IS05330-2, 1:3). Primary antibody was visualized using tyramide signal amplification linked to a specific fluorochrome from the mIF kit for each primary antibody. A stripping procedure based on microwaves was performed for each consecutive antibody staining. The stained slides were scanned using a Vectra® 3 multispectral microscope (Akoya Bioscience). From each slide, Vectra automatically captured the fluorescent spectra from 420 to 720 nm at 20-nm intervals with the same exposure time and then combined the captured images to create a single stack image that retained the particulate spectral signature of all markers. These data were analyzed using InForm 2.6 software.

Cell culture, cell proliferation assays, aromatase activity assays, CYP19A1 siRNA transfection, isolation of PBMCs and their coculture with colon cancer cells, flow cytometry analyses for tumor cell death and CD8^+^ T cell function, western blot analyses for CYP19A1 and PD-L1 expression, tumor models and therapeutic efficacy study, and statistical analysis were described in Supplementary Materials and Methods.

## Results

### Differential expression analysis of LMGs and functional annotation

**Fig. 1:**
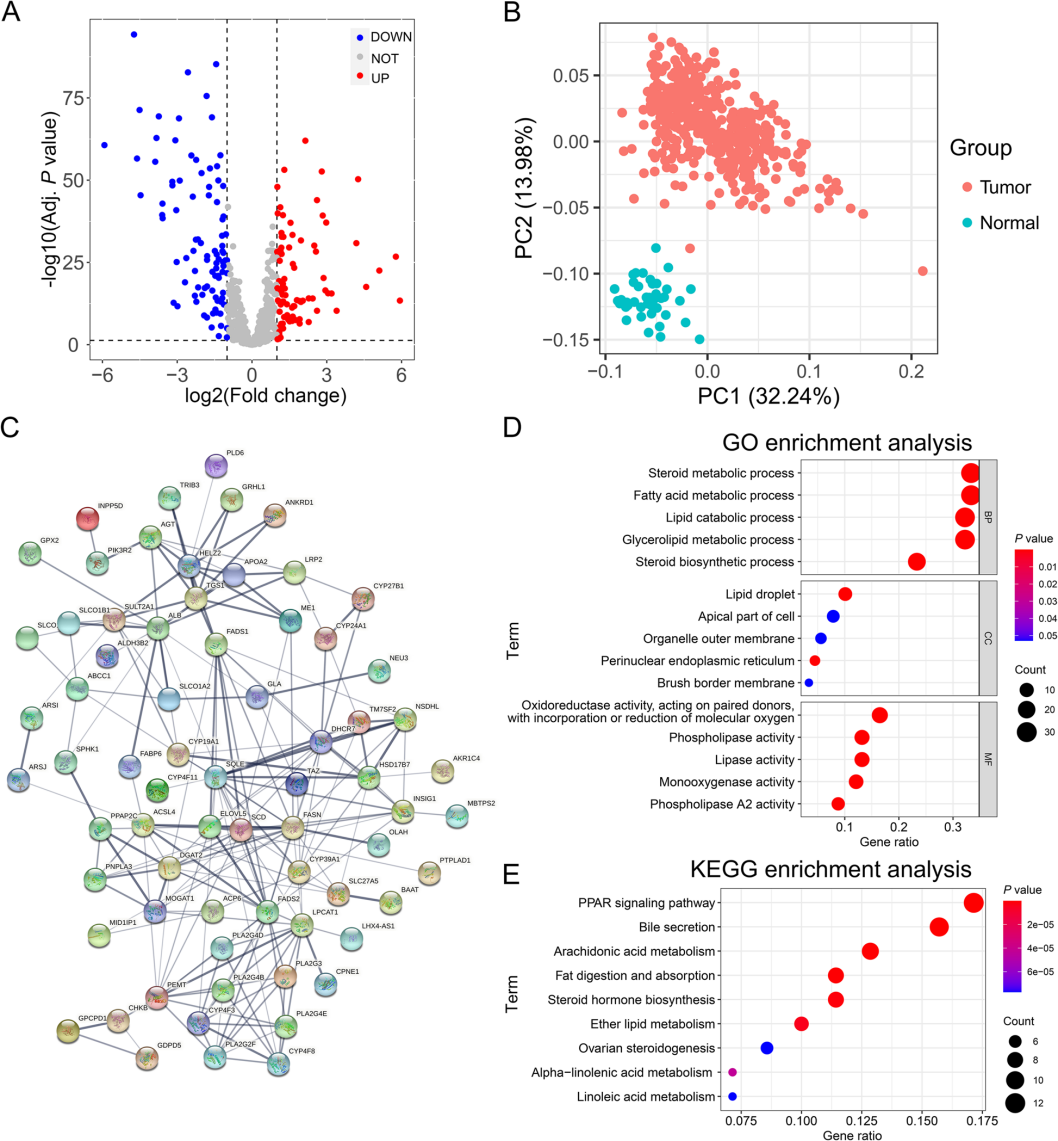
Differential expression analysis of LMGs and functional annotation in colon cancer. **A** Volcano plot of the differentially expressed lipid metabolism-related genes (LMGs) analysis in the colon cancer tissues (*n* = 453) and adjacent normal tissues (*n* = 41). Red/blue dots represent upregulated/downregulated genes according to the criteria: adjusted *P*-value < 0.05 and |log2 (fold change) |> 1. **B** Principal components analysis (PCA) based on the differentially expressed LMGs. **C** Protein–protein interaction network of differentially expressed lipid metabolism-related genes. **D** Gene ontology (GO) enrichment analysis of the differentially expressed LMGs. “BP” stands for “biological process”, “CC” stands for “cellular component” and “MF” stands for “molecular function”. **E** Kyoto Encyclopedia of Genes and Genomes (KEGG) enrichment analysis of the differentially expressed LMGs. The color represents the statistical significance of the term. The size indicates the counts of enriched genes

Expression data and clinical information of four cohorts were downloaded from the TCGA and GEO databases. The TCGA cohort consisted of 453 colon cancer and 41 normal samples, and was used for differential expression analysis. Seven hundred thirty-one LMGs were screened, and 178 LMGs were differentially expressed with 86 upregulation and 92 downregulation between colon cancer and normal samples (Fig. 1A). PCA revealed an obvious difference in a distinct cluster of these differentially expressed LMGs between the colon cancer tissues and adjacent normal tissues (Fig. 1B). The hub genes including CYP19A1 were identified by the protein–protein interaction network of differentially expressed LMGs in the colon cancer (Fig. 1C). Next, GO and KEGG pathway annotation analyses were performed to explore the biological functions of the differentially expressed LMGs in the colon cancer. GO analysis showed that these LMGs were enriched in the basic biological processes, including steroid metabolism, fatty acid metabolism, lipid catabolism and glycerolipid metabolism, and the most highly enriched terms for the cellular component and molecular function were lipid droplet and oxidoreductase activity, respectively (Fig. 1D). KEGG analysis showed that 178 LMGs were primarily related to AA metabolism, PPAR signaling pathway and bile secretion (Fig. 1E). These results suggest significant alterations of LMGs in human colon cancer.

### Construction and verification of the LMrisk

**Fig. 2:**
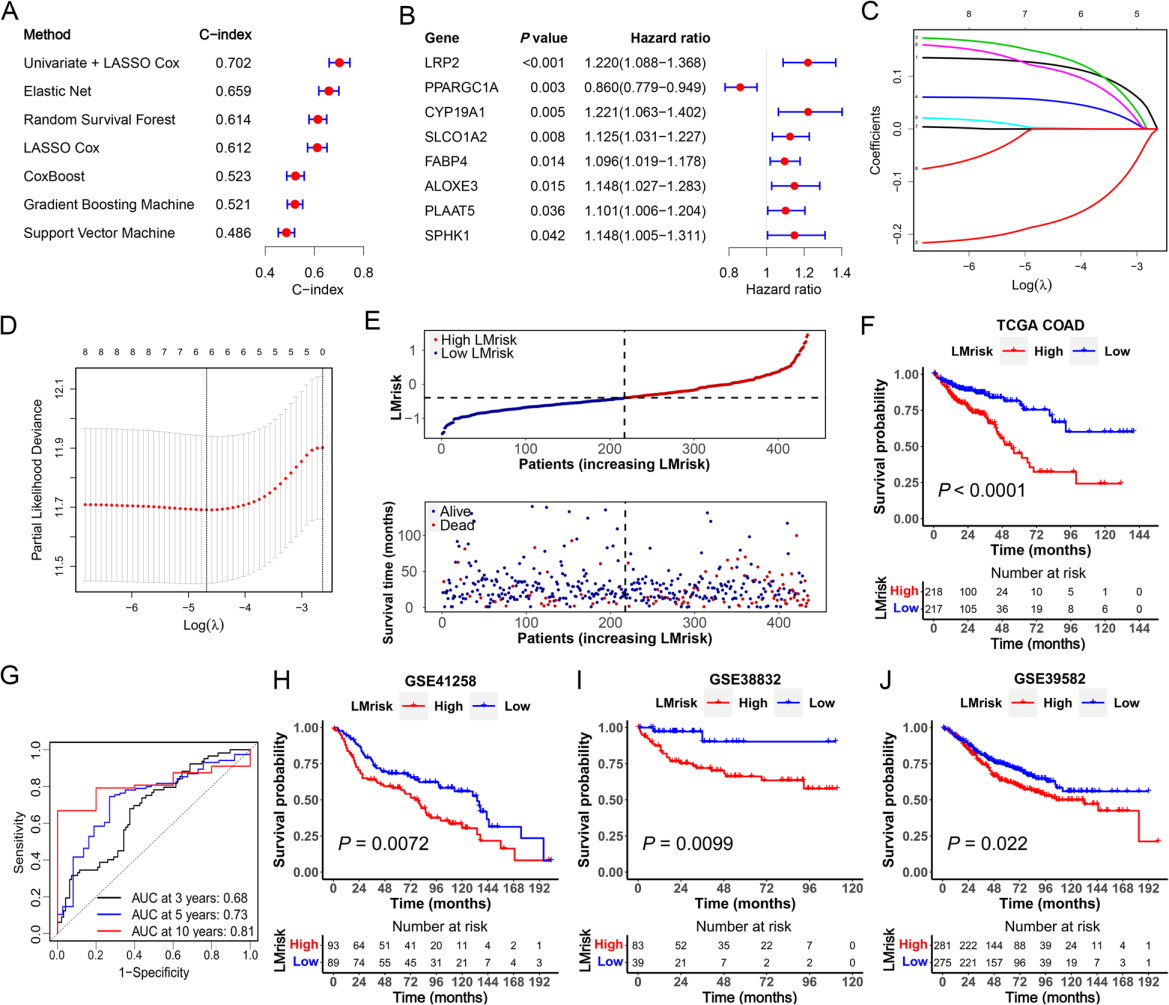
Construction and verification of a lipid metabolism-related prognostic risk core (LMrisk). **A** C-index of prognostic models constructed by multiple machine learning methods. **B** Forest plot of prognostic-related LMGs based on univariate Cox regression analysis. **C** The least absolute shrinkage and selection operator (LASSO) coefficient profiles of prognostic-related differentially expressed lipid metabolism-related genes (LMGs). **D** Tuning parameter (λ) selection in the LASSO model used ten-fold cross-validation based on the minimum criteria. The dotted vertical lines were drawn at the optimal values using the minimum criteria and one standard error of the minimum criteria. **E** Survival status and time distribution of patients in the high LMrisk and low LMrisk groups. **F** Kaplan–Meier survival curves for colon cancer patients according to the LMrisk in TCGA database. **G** Time-dependent receiver operating characteristic (ROC) curve analysis at 3, 5 and 10 years showing the area under curve values (AUC) for overall survival. **H**–**J** Kaplan–Meier survival curves for colon cancer patients according to the LMrisk in the GSE41258 (*n* = 182), GSE38832 (*n* = 122) and GSE39582 (*n* = 556) datasets

To develop prognostic models in the TCGA colon cancer cohort, machine learning methods such as random survival forest, CoxBoost, support vector machine, gradient boosting machine, elastic net and LASSO-Cox were employed. A univariate combined LASSO-Cox regression model with the maximum C-index (Fig. 2A) was used for subsequent analysis. Univariate Cox regression analysis revealed that the LRP2, CYP19A1, SLCO1A2, FABP4, ALOXE3, PLAAT5 and SPHK1 were risk genes with hazard ratio (HR) > 1, while PPARGC1A was protective gene with HR < 1 among 178 differentially expressed LMGs (Fig. 2B). By using LASSO-Cox regression analysis to further reduce overfitting, the six LMGs, including LRP2, CYP19A1, SLCO1A2, FABP4, ALOXE3 and PPARGC1A, were identified to construct the optimal lipid metabolism-related prognostic signature (Fig. 2C and D). The expression levels and LASSO coefficients of six LMGs were extracted to calculate the LMrisk for each patient with the following formula: LMrisk = (0.124 × LRP2 expression) + (0.145 × CYP19A1 expression) + (0.0551 × SLCO1A2 expression) + (0.00237 × FABP4 expression) + (0.117 × ALOXE3 expression) + (-0.182 × PPARGC1A expression). Based on the median LMrisk, the patients were assigned to low LMrisk (*n* = 217) and high LMrisk groups (*n* = 218). We observed more deaths and shorter survival time in the colon cancer patients with the high LMrisk as compared with the low LMrisk (Fig. 2E and F). Next, time-dependent ROC curves were used to evaluate the predictive value of the LMrisk. As shown in Fig. 2G, the AUC of LMrisk at 3, 5 and 10 years were 0.68, 0.73 and 0.81, respectively. Consistently, Kaplan–Meier survival curves indicated that the survival time was shorter in the high LMrisk group than in the low LMrisk group in the GSE41258 (*P* = 0.0072), GSE38832 (*P* = 0.0099) and GSE39582 (*P* = 0.022) datasets (Fig. 2H–J). To validate the predictive ability of the LMrisk on colon cancer prognosis, we conducted a stratified analysis based on clinicopathological features including age, gender, TNM stage, N stage, M stage and T stage in the high and low LMrisk groups. Kaplan–Meier survival analyses revealed that the high-risk group had worse overall survival compared to the low LMrisk group in different strata of clinical characteristics including younger (< 66 years) or older (≥ 66 years), male or female, stage I–II or stage III–IV, N0 or N1–N2, M0 or M1 and T3–T4 patients (Fig. S1). These results suggest that the LMrisk has good robustness for predicting prognosis of colon cancer.

To identify the factors affecting the survival of patients with colon cancer, we analyzed the prognostic value of the LMrisk and clinicopathological parameters in the TCGA dataset. As shown in Fig. S2A, the LMrisk, in addition to clinical factors (age, T stage, N stage, M stage and TNM stage), was closely associated with survival outcomes. Multivariate analysis manifested that the LMrisk, age and TNM stage were independent prognostic indicators (Fig. S2B). The AUC of LMrisk was larger than other indicators including TNM stage, suggesting that the LMrisk has a better ability to predict prognosis in the colon cancer (Fig. S2C). The LMrisk was significantly correlated with the age, T stage, N stage, M stage and TNM stage. We also observed relatively low expression of PPARGC1A and high expression of CYP19A1, FABP4, ALOXE3, LRP2 and SLCO1A2 in the patients with high LMrisk (Fig. S2D). The patients had higher LMrisk in stage III–IV than in stage I–II (*P* = 0.023), and in T3–T4 than in T1–T2 (*P* = 0.016). The patients with lymph node metastasis (N1–N2) or distant metastasis (M1) had higher LMrisk than those without lymph node metastasis (N0) or distant metastasis (M0) (Fig. S2E). These data suggest that the LMrisk is an independent prognostic risk factor in the colon cancer.

### Construction and validation of a prognostic nomogram based on LMrisk

To predict overall survival probabilities of colon cancer patients, a prognostic nomogram was established by integrating the LMrisk with age and TNM stage in the TCGA dataset (Fig. S3A). We used calibration curve to verify the accuracy of the prediction model, and found that the predicted probability of 3- and 5-year survival fitted well with the observed survival (Fig. S3B and C). The AUC values of nomogram were higher than those of TNM stage in 3 and 5 year (Fig. S3D and E). The nomogram also showed satisfactory discrimination and calibration with a C-index of 0.79 (95% confidence interval: 0.74–0.84). These data suggest that our nomogram presents superior prognostic value than that of TNM stage in colon cancer.

### The association of LMrisk with immune cell infiltration

**Fig. 3:**
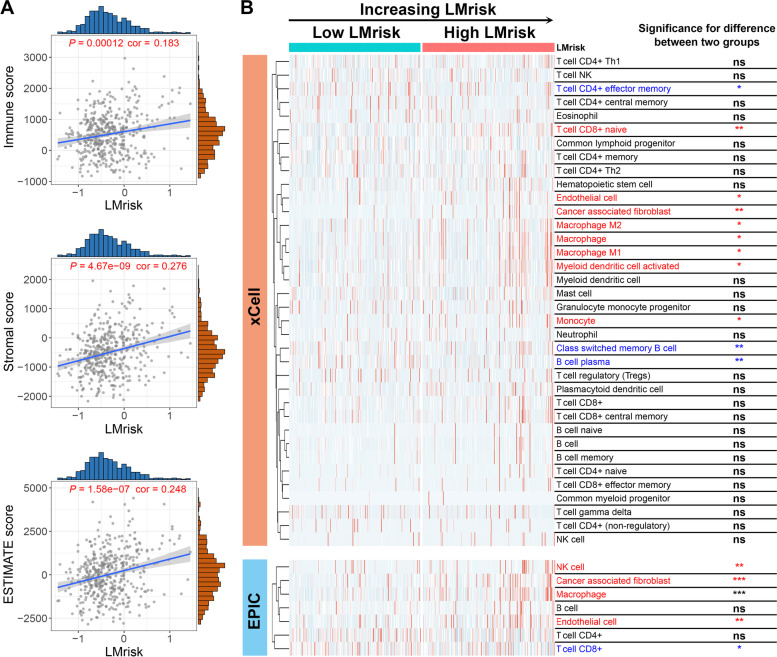
Correlation between the lipid metabolism-related prognostic risk core (LMrisk) and immune microenvironment scores and infiltration levels of immune cells and stromal cells. **A** Correlations of the LMrisk with immune score, stromal score and ESTIMATE score calculated by ESTIMATE algorithm. **B** The heatmap showed the normalized infiltrations of immune and stromal cells. Blue/red represents cells with lower/higher infiltration in the high LMrisk group than in the low LMrisk group. ^*^, *P* < 0.05; ^**^, *P* < 0.01; ^***^, *P* < 0.001; ns, not significant

The tumor immune microenvironment innately modulates tumor progression [28]. Thus, the ESTIMATE algorithm was used to evaluate the immune score and stromal score of colon cancer patients. Figure 3A showed that the LMrisk was positively correlated with the immune score, stroma score and ESTIMATE score in the TCGA dataset. Next, immune cell and stromal cell infiltration in colon cancer tissues were inferred by xCell and EPIC algorithms. We observed less infiltration of CD8^+^ T cells, but more proportions of immunosuppressive cells including macrophages, monocytes, CAFs and endothelial cells in the high LMrisk group compared to the low LMrisk group (Fig. 3B). These data confirm that the infiltration of CD8^+^ T cells is decreased but the infiltration of immunosuppressive cells is increased in the colon cancer with high LMrisk.

### Association of the LMrisk with immunotherapy response in colon cancer

**Fig. 4:**
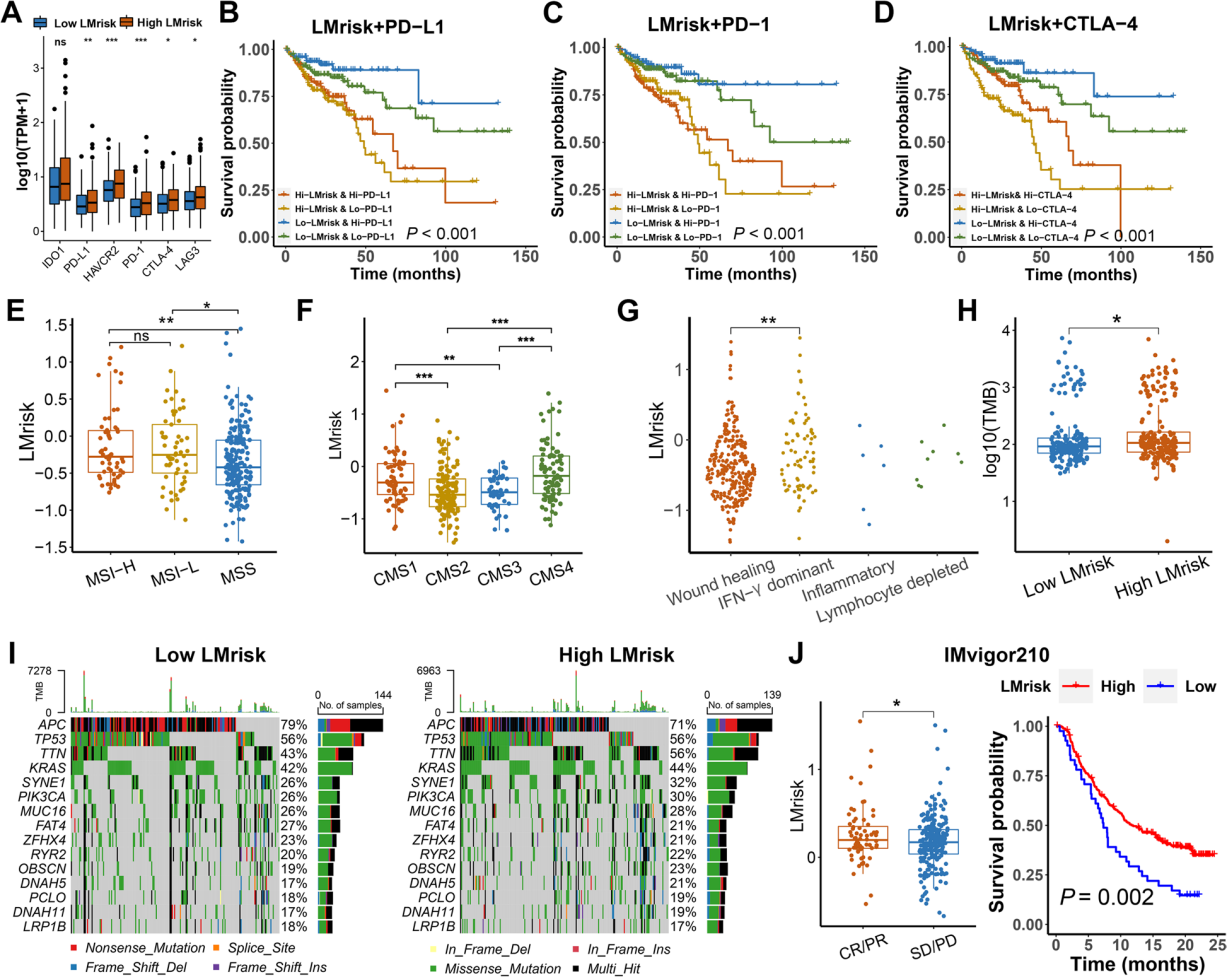
The lipid metabolism-related prognostic risk core (LMrisk) predicts therapeutic responses of immune checkpoint inhibitors in colon cancer. **A** Immune checkpoint genes expression levels in the high and low LMrisk groups in TCGA. **B**–**D** Kaplan–Meier survival curves of overall survival among four groups stratified by the LMrisk and expression of PD-L1, PD-1 and CTLA-4. **E** The LMrisk of colon cancer patients with microsatellite instability-high (MSI-H), microsatellite instability-low (MSI-L) and microsatellite stability (MSS). **F** The LMrisk of colon cancer patients with different CMS. **G** The LMrisk of colon cancer patients in four immune subtypes. **H** The TMB in the high and low LMrisk groups. **I** Top 15 mutated genes were illustrated in the high and low LMrisk groups. **J** The LMrisk of the patients with CR/PR and SD/PD. Kaplan–Meier survival curves for the patients according to the LMrisk in IMvigor210 cohort. CR, complete response; PR, partial response; SD, stable disease; PD, progressive disease. ^*^, *P* < 0.05; ^**^, *P* < 0.01; ^***^, *P* < 0.001; ns, not significant

The TME includes immune checkpoint regulators as well as inflammatory mediators besides immune cells [13]. To understand the molecular mechanisms altering immunotherapy responsiveness, we evaluated the association of the LMrisk with immunotherapy response in colon cancer. As shown in Fig. 4A, PD-1, PD-L1, CTLA-4, LAG3 and HAVCR2 (TIM3) were significantly overexpressed in the high LMrisk group than in the low LMrisk group. The patients with low LMrisk and high PD-L1 had better survival than those with high LMrisk and high PD-L1, and patients with low LMrisk and low PD-L1 had better survival than those with high LMrisk and low PD-L1 (Fig. 4B). Similar results were obtained in the PD-1 and CTLA-4 stratifying groups (Fig. 4C and D). MSI status, consensus molecular subtypes (CMS) heterogeneity and TMB display different immune landscapes in the TME, and therefore are used as predictive biomarkers for response of ICIs [29–32]. Accordingly, we compared the LMrisk between different MSI status, and found that the LMrisk was higher in the colon cancer with MSI-H and MSI-L as compared with MSS (Fig. 4E). The LMrisk was higher in CMS1 (immune activated phenotype) or CMS4 (immune inflamed phenotype) than in CMS2 (immune desert phenotype) and CMS3 (immune excluded phenotype) (Fig. 4F). A previous study shows that, in six immune subtypes, IFN-γ dominant subtype has better ICI outcomes [33, 34]. We observed the significantly increased LMrisk in IFN-γ dominant subtype than in wound healing subtype, though there was no significant difference of LMrisk between inflammatory and lymphocyte depleted subtypes (Fig. 4G). The TMB was significantly increased in the patients with high LMrisk as compared with low LMrisk (Fig. 4H). Maftools analysis results showed that TTN gene mutated more frequently in the high LMrisk than in the low LMrisk group (Fig. 4I). Because of unavailable datasets for colon cancer patients treated with immune checkpoint blockade, we analyzed publicly available datasets from urothelial cancer patients treated with anti-PD-1 therapy. As shown in Fig. 4J, the high LMrisk was correlated with a complete or partial response (CR/PR), while low LMrisk correlated with stable disease or progressive disease (SD/PD) when the patients received anti-PD-1 therapies. The high LMrisk also predicted significant improvement in overall survival for the patients received anti-PD-1 therapy compared with the low LMrisk. These results indicate that the LMrisk is a potential predictive biomarker of immunotherapeutic response.

### CYP19A1, a risk factor of the LMrisk model, is associated with immune signature

**Fig. 5:**
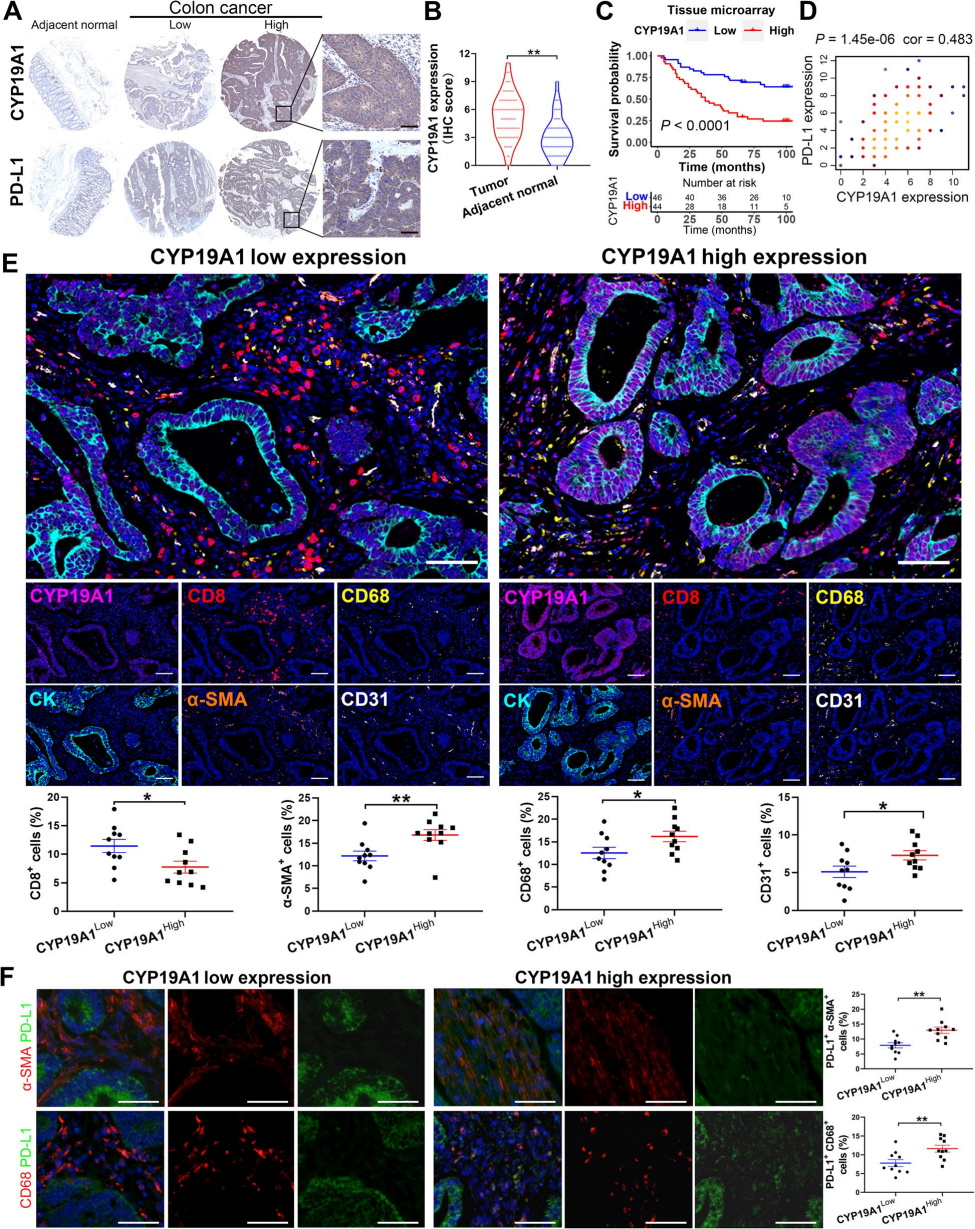
CYP19A1 is highly expressed in colon cancer and is correlated with poor prognosis, PD-L1 level and the infiltration levels of immune cells and stromal cells. **A** Representative images of immunohistochemistry (IHC) staining of CYP19A1 and PD-L1 protein in human colon cancer tissue microarrays. **B** Statistical analysis of CYP19A1 IHC score in colon tumor tissue microarray (*n* = 90). **C** Kaplan–Meier survival curves for colon cancer patients according to the IHC score of CYP19A1. **D** Correlation analysis of CYP19A1 and PD-L1 protein expression (n = 90). **E** Representative images of multiplex immunofluorescence in CYP19A1-low (*n* = 10) and CYP19A1-high (*n* = 10) human colon cancer tissues. Quantification of CD8^+^ T cells, CD31^+^ endothelial cells, CD68^+^ macrophages and α-SMA^+^ CAFs as a proportion of total cells. **F** The correlations between CYP19A1 expression and PD-L1 level in CAFs and macrophages were analyzed by immunofluorescence. Scale bars, 50 μm. ^*^, *P* < 0.05; ^**^, *P* < 0.01

Among the differentially expressed lipid metabolism genes in colon cancer and adjacent normal tissues, CYP19A1 is a key molecular node and has the largest prognostic hazard ratio (Figs. 1C and 2B). Therefore, CYP19A1, a risk factor of the LMrisk model, was used for subsequently experimental verification. We found that CYP19A1 gene was highly expressed in the colon cancer tissues compared to the adjacent normal tissues in the Gene Expression Profiling Interactive Analysis (GEPIA) webserver (Fig. S4A). CYP19A1 gene expression was a strong prognostic risk factor in the colon cancer (Fig. S4B), and positively correlated with PD-L1 expression in the GEPIA webserver (Fig. S4C). Subsequently, we conducted functional verification by using a tissue microarray of human colon cancer. Similarly, CYP19A1 protein expression was significantly increased in human colon cancer tissues (Fig. 5A and B), and positively correlated with PD-L1 expression (Fig. 5D). CYP19A1 protein expression in the colon cancer tissues was negatively correlated with overall survival (Fig. 5C), and positively associated with N stage, and an independent prognostic parameter of overall survival (Tables S1 and S2). Next, mIF staining was performed to explore the relationship between CYP19A1 expression and immune landscape in the colon cancer. We observed an apparent colocalization of CYP19A1 with CK (tumor cell marker), indicating that CYP19A1 is mainly expressed in tumor cells. The infiltration of CD8^+^ T cells was more but the proportions of CD68^+^ cells (macrophages), α-SMA^+^ cells (CAFs) and CD31^+^ cells (endothelial cells) were fewer in human colon cancer tissues with low CYP19A1 expression as compared with high CYP19A1 expression (Fig. 5E). We analyzed PD-L1 expression in the same biopsies, and found that PD-L1^+^ CAFs and macrophages were significantly higher in CYP19A1 high expression group as compared with low CYP19A1 expression group (Fig. 5F). These data indicate that high CYP19A1 expression leads to poor prognosis, accompanied by the low infiltration level of CD8^+^ T cells and high infiltration levels of immunosuppressive cells in colon cancer.

### CYP19A1 inhibition potentiates CD8^+^ T cell-mediated anti-tumor immune response in vitro

**Fig. 6:**
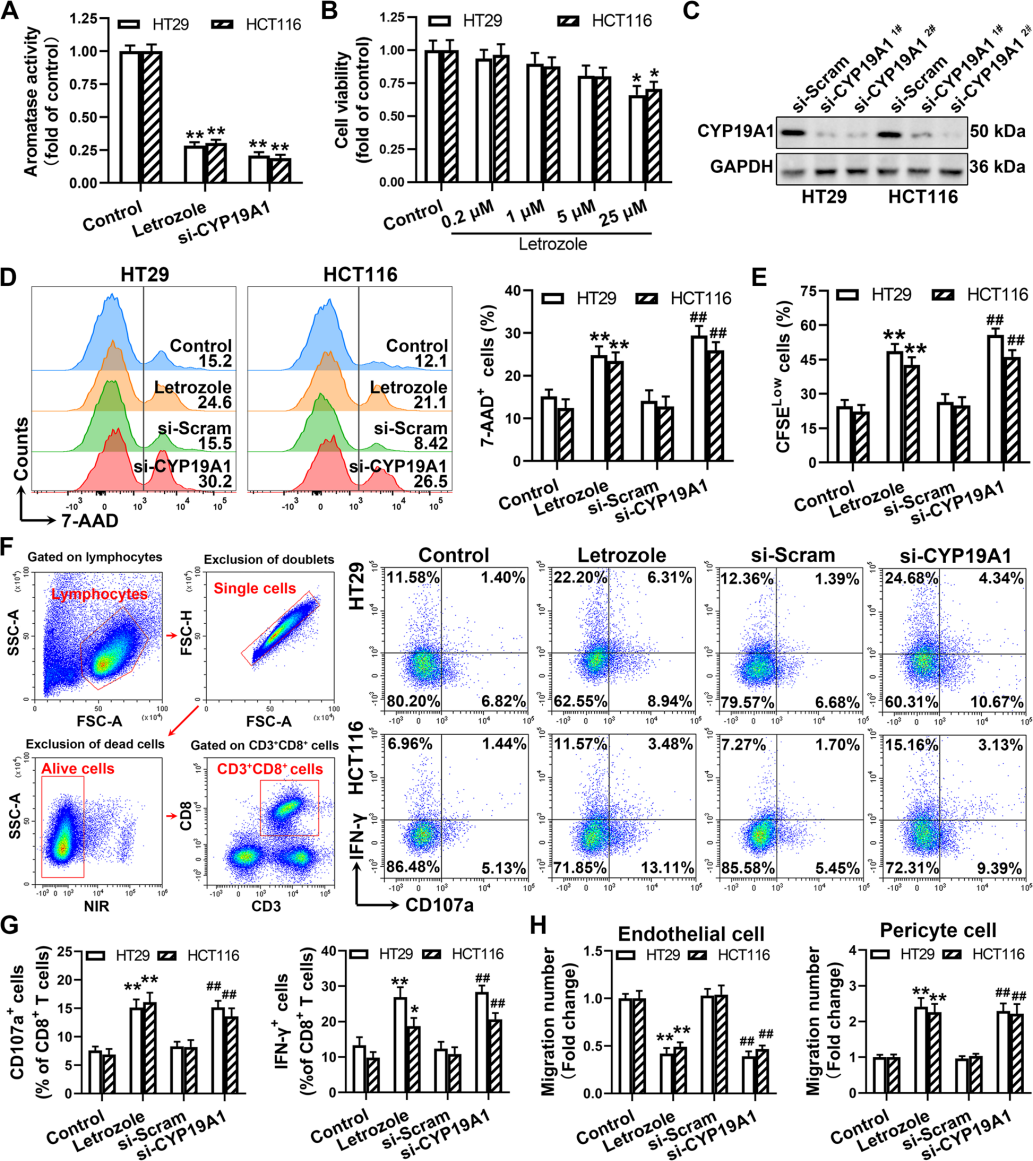
CYP19A1 inhibition potentiates CD8^+^ T cell-mediated antitumor immunity in vitro coculture model. **A** The HT29 or HCT116 cells were treated with the letrozole or vehicle for 24 h. Aromatase activity was evaluated by culturing cells with testosterone and measuring estradiol levels in the culture medium with enzyme-linked immunosorbent assay. **B** The viabilities of HT29 and HCT116 cells were determined by MTT assay. **C** The level of CYP19A1 protein expression was measured in HT29 and HCT116 cells transfected with CYP19A1 siRNA (si-CYP19A1) or si-Scram. Human PBMCs were cocultured with the letrozole (5 μM) or si-CYP19A1-treated HT29 cells or HCT116 cells for 24 h. **D** Tumor cells pre-incubated with anti-EpCAM antibody were stained by 7-AAD, and then the proportion of EpCAM^+^ 7-AAD^+^ cells were analyzed by flow cytometry. **E** Carboxyfluorescein succinimidyl ester (CFSE) dilution was used to measure the proliferation of CD8^+^ T cells. **F** and **G** The percentage of IFN-γ^+^ or CD107a^+^ CD8^+^ cells were determined by flow cytometry. **H** Transwell migration analysis of human brain vascular pericytes (HBVP) and human umbilical vein endothelial cells (HUVEC) treated with the conditioned medium (CM) from the above treated-HT29 cells and HCT116 cells. The number of the migrated cells was counted. The values are presented as the mean ± standard error of the mean, *n* = 5. ^*^, *P* < 0.05; ^**^, *P* < 0.01 *vs.* control. ^##^, *P* < 0.01 *vs.* si-Scram

To gain insight into the role of CYP19A1 in tumor immunity, the inhibitor (letrozole) and siRNAs of CYP19A1 were used in the in vitro co-culture of human HT29 and HCT116 cells with PBMCs. We found that the letrozole (5 μM) significantly decreased aromatase activity in human HT29 and HCT116 cells (Fig. 6A) without affecting their proliferation (Fig. 6B). The CYP19A1 siRNAs significantly inhibited CYP19A1 expression (Fig. 6C). Unsurprisingly, CYP19A1 inhibition by the letrozole or siRNA in tumors increased the proportion of 7-AAD^+^ tumor cells after co-culture with PBMCs (1.5- and 2-fold, respectively) (Fig. 6D). The letrozole or CYP19A1 siRNA-treated HT29 and HCT116 cells significantly increased CD8^+^ T cell proliferation and the proportion of CD8^+^ CD107a^+^ and CD8^+^ IFN-γ^+^ T cells (Fig. 6E–G), and promoted pericyte cell migration and reduced endothelial cell migration (Fig. 6H). These data indicate that CYP19A1 inhibition in colon cancer cells enhances CD8^+^ T cell-mediated antitumor immunity.

### CYP19A1 inhibits CD8^+^ T cells through upregulation of PD-L1, IL-6 and TGF-β

**Fig. 7:**
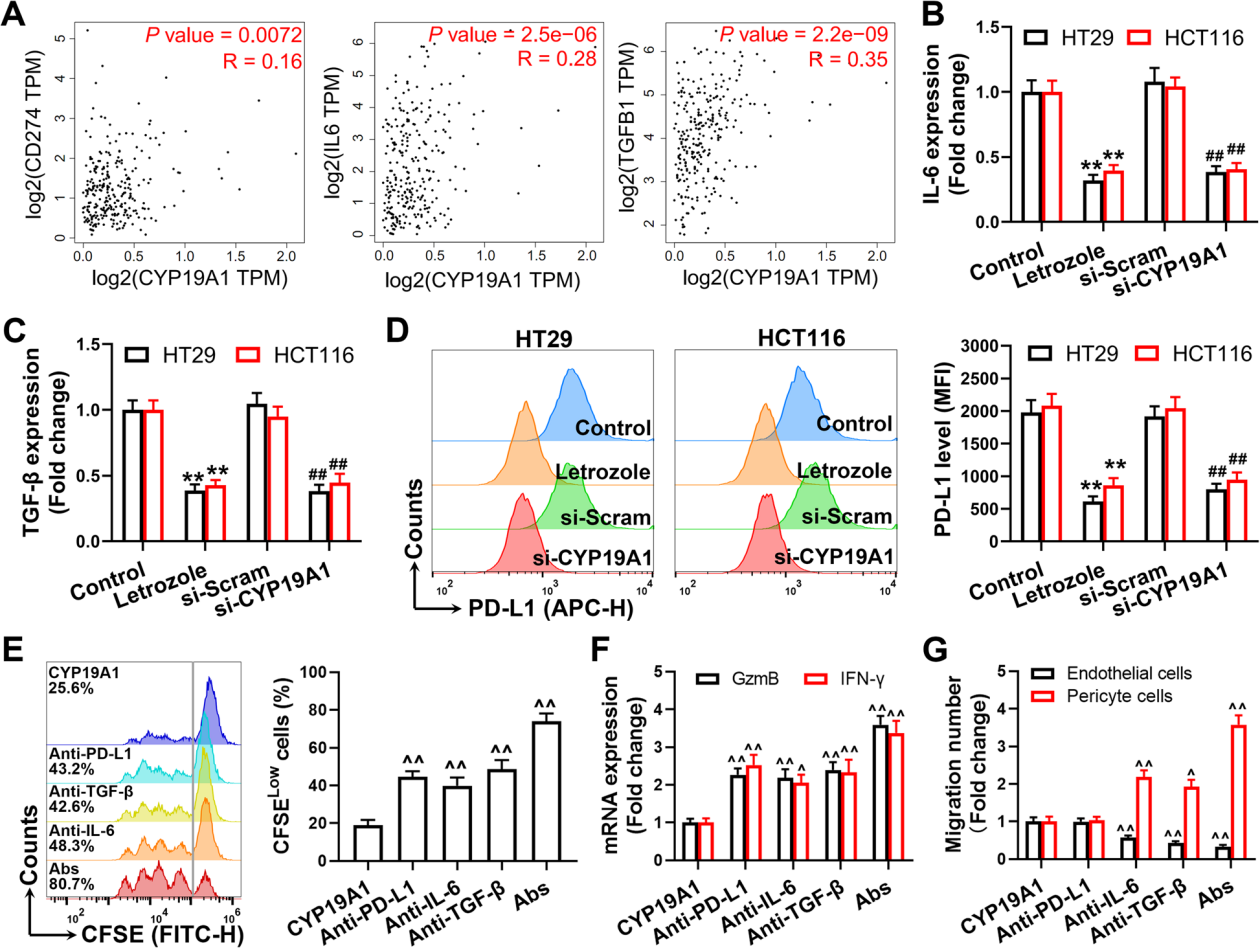
CYP19A1 inhibits CD8^+^ T cells through upregulation of PD-L1, IL-6 and TGF-β. **A** Correlations of CYP19A1 with PD-L1, IL-6 and TGF-β expression levels in GEPIA database. **B** and **C** IL-6 and TGF-β expression levels in HT29 and HCT116 cells were measured by qPCR. **D** PD-L1 expression level was determined by flow cytometry. **E** CFSE dilution was used to measure the proliferation of CD8^+^ T cells. **F** GzmB and IFN-γ in CD8^+^ T cells were determined by qPCR. **G** Transwell migration analysis of endothelial cells and pericyte cells treated with the conditioned medium from the above treated-HT29 cells. The values are presented as the mean ± standard error of the mean, *n* = 5. ^**^, *P* < 0.01 *vs.* control. ^##^, *P* < 0.01 *vs.* si-Scram. ^^^, *P* < 0.01; ^^^^, *P* < 0.01 *vs.* CYP19A1

Tumor cells upregulate the expression of immune checkpoint molecules or secrete cytokines to induce abnormal angiogenesis, thereby promoting tumor immune escape [35, 36]. We found that CYP19A1 expression level was significantly positively correlated with PD-L1, IL-6 and TGF-β levels in the TCGA colon cancer dataset (Fig. 7A). Next, we verified the factors derived from tumor cells in HT29 and HCT116 colon cancer cells, and comfirmed that CYP19A1 inhibition significantly reduced PD-L1, IL-6, and TGF-β expression in tumor cells (Fig. 7B–D). Neutralizing antibodies against PD-L1, IL-6 or TGF-β partially ameliorated the effects of CYP19A1 overexpression on the proliferation and effector function of CD8^+^ T cells (Fig. 7E and F). Neutralizing IL-6 and TGF-β also promoted pericyte cell migration and inhibited endothelial cell migration (Fig. 7G). Importantly, combined blockade of PD-L1, IL-6 and TGF-β exerted better efficacy than single blockade (Fig. 7E–G). These data highlight compelling evidence that CYP19A1 inhibits the proliferation and effector function of CD8^+^ T cells by upregulating PD-L1, IL-6 and TGF-β expression in colon cancer cells.

### CYP19A1/estradiol mediates immunosuppression via GPR30-Akt signaling

**Fig. 8:**
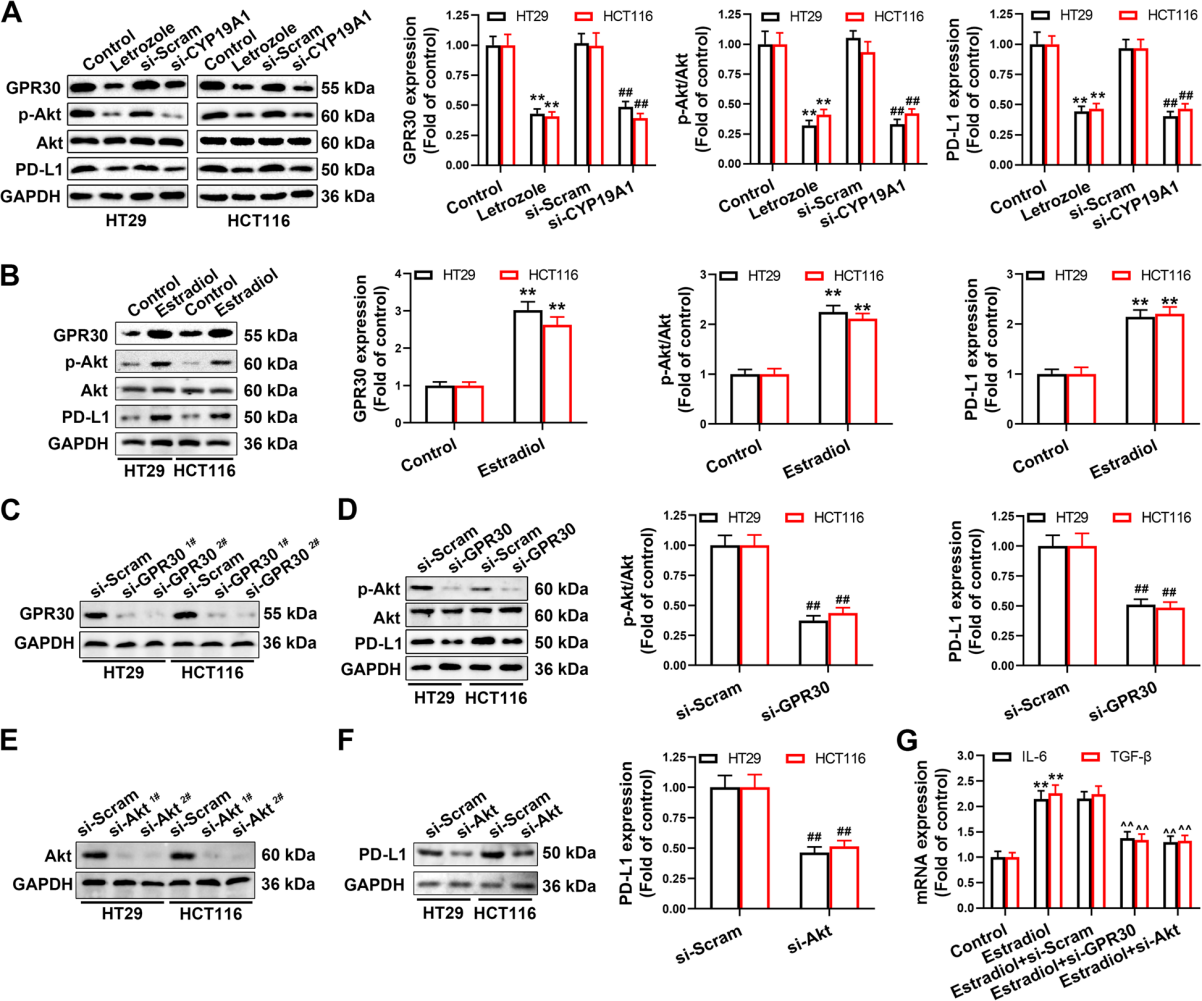
CYP19A1/estradiol mediates immunosuppression via GPR30-Akt signaling. **A** GPR30, p-Akt and PD-L1 protein levels in HT29 and HCT116 cells treated with letrozole and si-CYP19A1. **B** GPR30, p-Akt and PD-L1 protein levels in HT29 and HCT116 cells treated with estradiol. **C** GPR30 protein level in HT29 and HCT116 cells transfected with si-GPR30. **D** PD-L1 and p-Akt protein levels in HT29 and HCT116 cells transfected with si-GPR30. **E** Akt protein level in HT29 and HCT116 cells transfected with si-Akt. **F** PD-L1 protein level in HT29 and HCT116 cells transfected with si-Akt. **G** TGF-β and IL-6 mRNA levels in HT29 cells of indicated groups. The values are presented as the mean ± standard error of the mean, *n* = 5. ^*^, *P* < 0.05; ^**^, *P* < 0.01 *vs.* control. ^#^, *P* < 0.05; ^##^, *P* < 0.01 *vs.* si-Scram. ^^^, *P* < 0.05; ^^^^, P < 0.01 *vs.* estradiol + si-Scram

Activation of G-protein coupled estrogen receptor 30 (GPR30) promotes colon cancer growth through mediating estrogenic activity in colon cancer cells [37], and Akt signaling is one of the key downstream pathways of GPR30 in cancer progression [38]. We discovered that CYP19A1 inhibition by letrozole or si-CYP19A1 downregulated GPR30, p-Akt and PD-L1 in HCT116 and HT29 colon cancer cells (Fig. 8A), which were greatly enhanced by exogenous addition of estradiol (Fig. 8B). GPR30 knockdown decreased p-Akt and PD-L1 levels (Fig. 8C and D), and Akt knockdown also inhibited PD-L1 protein expression in HCT116 and HT29 colon cancer cells (Fig. 8E and F). Exogenous supplementation of estradiol increased IL-6 and TGF-β expression in HCT116 and HT29 colon cancer cells (Fig. 8G). Importantly, GPR30 or Akt siRNA reversed the increased expression of IL-6 and TGF-β in HCT116 and HT29 colon cancer cells in response to estradiol stimulation (Fig. 8G). These results imply that GPR30-Akt signaling is crucial for CYP19A1/estradiol-mediated immunosuppression in colon cancer.

### Nanoparticle-encapsulated CYP19A1 siRNA synergizes with anti-PD-1 therapy

**Fig. 9:**
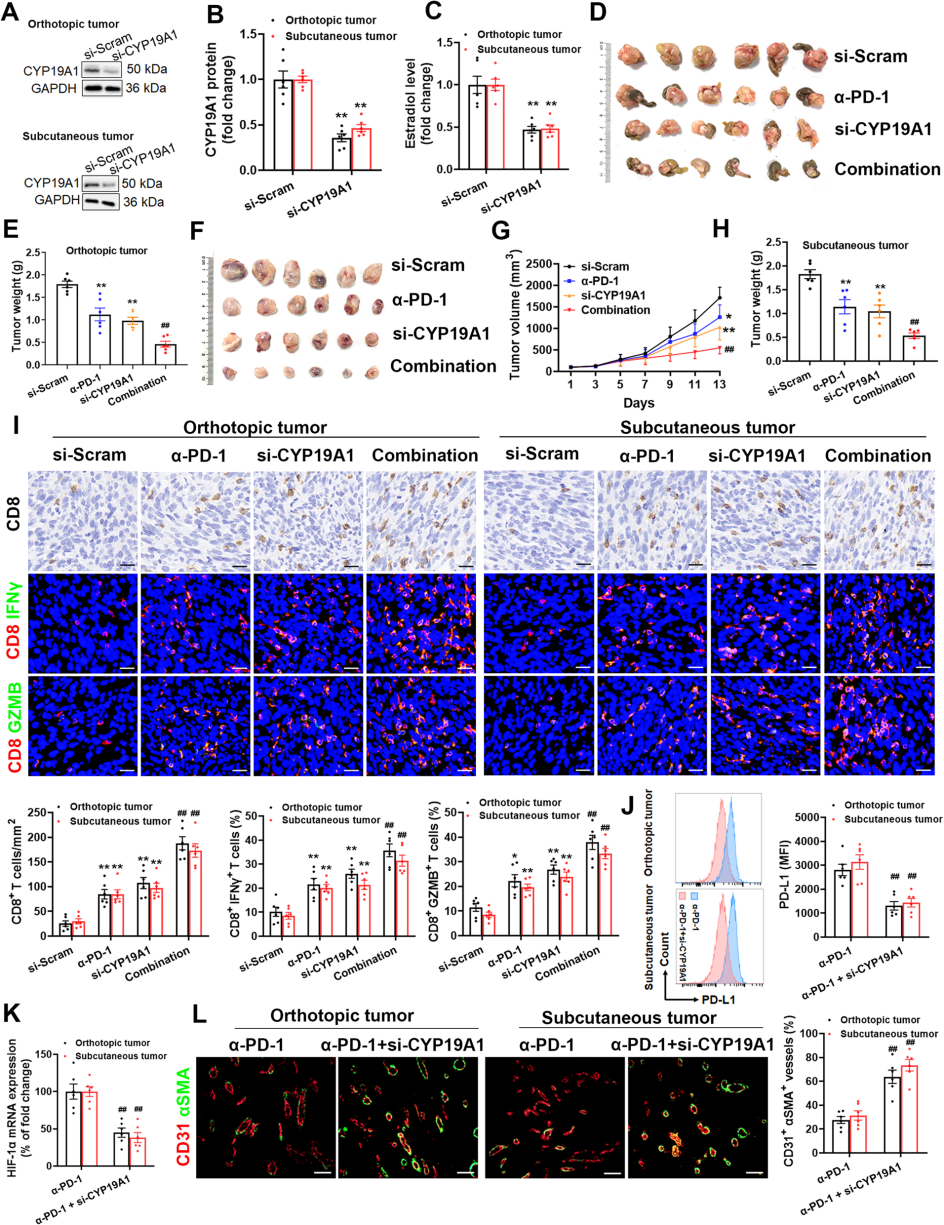
Nanoparticle-encapsulated CYP19A1 siRNA synergizes with anti-PD-1 immunotherapy. **A** and **B** CYP19A1 protein level in orthotopic and subcutaneous colon tumor tissues. **C** Intratumoral estradiol level. **D** Representative ex vivo images of colon with an orthotopic tumor in the cecum. **E** Average orthotopic tumor weight. **F** Photographs of excised subcutaneous MC38 tumors. **G** Tumor growth curves in subcutaneous MC38 tumor-bearing mice. **H** Average subcutaneous tumor weight. **I** Representative immunohistochemistry (IHC) staining of CD8 and double staining for CD8 (red) and IFNγ (green) or GzmB (green) and their quantitative analyses in the MC38 tumors. Scale bar, 20 μm. **J** The level of PD-L1 protein in the tumor cells. **K** Hypoxia-inducible factor (HIF) -1α mRNA level. **L** Representative immunofluorescence staining and frequency of CD31 (red) and αSMA (green) in the MC38 tumor tissues. Scale bar, 50 μm. The values are presented as the mean ± standard error of the mean, *n* = 6. ^*^, *P* < 0.05; ^**^, *P* < 0.01 *vs*. si-Scram. ^##^, *P* < 0.01 *vs*. α-PD-1

The established hyaluronic acid-modified chitosan nanoparticles are recently shown to be effective for the systemic administration of siRNA to tumors [39, 40]. To explore the possibility of using CYP19A1 as a target for sensitizing anti-PD-1 therapy, the effect of nanoparticle-encapsulated CYP19A1 siRNA on PD-1 blockade therapy was investigated in the orthotopic and subcutaneous colon cancer models. We detected obvious decreases in CYP19A1 protein expression and estradiol production in siCYP19A1-treated MC38 tumor as compared with the si-Scram (Fig. 9A-C). Knockdown of CYP19A1 by specific siRNA significantly impeded growth of orthotopic and subcutaneous MC38 colon cancer, but its combination with anti-PD-1 treatment was far more effective than monotherapy (Fig. 9D-H). CYP19A1 knockdown boosted tumor-infiltrating CD8^+^ T cells and enhanced GzmB and IFNγ production, indicative of CD8^+^ T cells-elicited adaptive immunity against colon cancer, which were amplyfied in combination therapy (Fig. 9I). Additionally, the CYP19A1 siRNA remarkably inhibited PD-L1 expression on tumor cells (Fig. 9J), induced vascular normalization, as manifested by an obvious decrease in the hypoxia-inducible factor (HIF) -1α level and an increase in CD31^+^ αSMA^+^ cells number (Fig. 9K and L). Together, our results demonstrate that CYP19A1 inhibition drastically enhances anti-PD-1 therapy for colon cancer.

### Letrozole facilitates anti-PD-1 therapy by promoting CD8^+^ T cell-mediated anti-tumor immune response in murine models

**Fig. 10:**
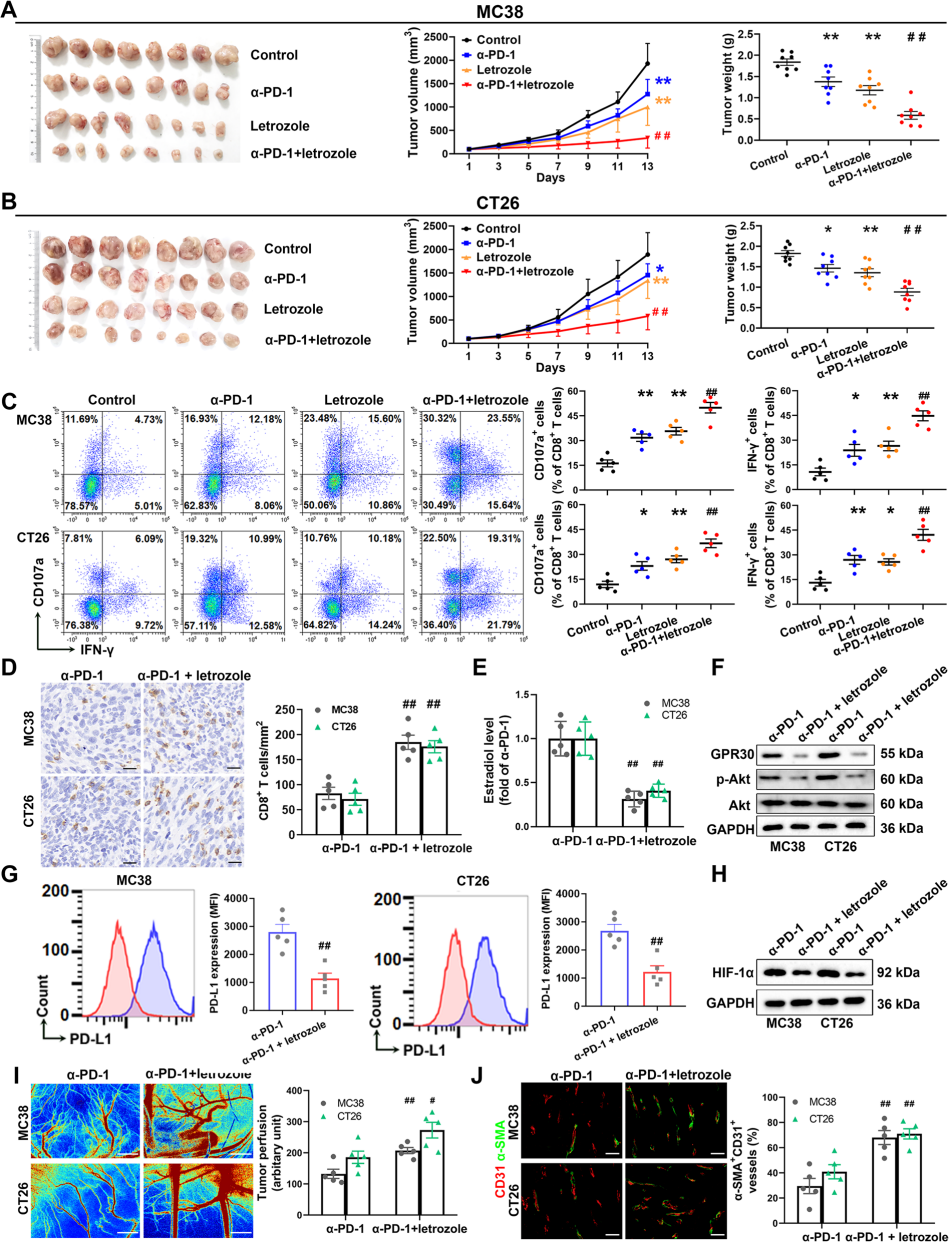
CYP19A1 inhibitor letrozole facilitates anti-PD-1 therapy in MC38 and CT26 mouse tumor models. **A** and **B** Tumor growth curves and tumor weight of C57BL/6 or BALB/c mice injected subcutaneously with MC38 or CT26 colon cells with the treatment of IgG control (IgG), letrozole, anti-PD-1 antibody (α-PD-1) alone or in combination with letrozole (*n* = 8). **C** Representative flow staining and frequency of CD107a^+^ CD8^+^ T cells and IFN-γ^+^ CD8^+^ T cells in tumor tissues of indicated groups. **D** The infiltration level of CD8^+^ T cells in tumor tissues was analyzed by immunohistochemistry. **E** Intratumoral estradiol level was detected by ELISA. **F** GPR30 and p-Akt/Akt levels were measured in MC38 and CT26 tumor tissues by western blot. **G** The expression level of PD-L1 on tumor cells was determined by flow cytometry. **H** Hypoxia-inducible factor (HIF) -1α level was measured in MC38 and CT26 tumor tissues by western blot. **I** Tumor perfusion was measured using a laser Doppler analyzer in the MC38 and CT26 tumor model. Scale bar, 2 mm. The quantitative analysis showed the relative levels of tumor perfusion in the tumors. **J** Immunofluorescence analysis of tumor vessel normalization. Scale bar, 50 μm. *n* = 5. The values are presented as the mean ± standard error of the mean. ^*^, *P* < 0.05; ^**^, *P* < 0.01 *vs*. control. ^#^, *P* < 0.05; ^##^, *P* < 0.01 *vs.* α-PD-1

Next, we further explored the possibility of using CYP19A1 as a target for sensitizing anti-PD-1 therapy, and found that the CYP19A1 inhibitor letrozole sensitized anti-PD-1 therapy in subcutaneous tumors derived from the MC38 and CT26 murine colon cancer (Fig. 10A and B). The combination of letrozole with PD-1 blockade promoted expression of IFNγ and CD107a and intratumoral infiltration of CD8^+^ T cells compared to the monotherapy (Fig. 10C and D). The combination treatment reduced GPR30 and p-Akt levels in MC38 and CT26 colon cancer tissues with an obvious decrease in estradiol production compared to the anti-PD-1 monotherapy (Fig. 10E and F). The letrozole inhibited PD-L1 expression in tumor cells (Fig. 10G), boosted tumor perfusion, promoted vascular normalization, and downregulated HIF-1α in CT26 and MC38 tumor (Fig. 10H–J). Similar results were obtained in the mice model of orthotopic MC38 colon cancer (Supplementary Fig. S5). The combination of letrozole with anti-PD-1 treatment did not result in any extra toxicity compared with the anti-PD-1 monotherapy (Supplementary Table S3). Overall, these findings strongly indicate that CYP19A1 inhibition greatly facilitates anti-PD-1 therapy for colon cancer.

## Discussion

Previous studies have shown that the alterations in lipid metabolism including cholesterol metabolism and AA metabolism affect immunotherapeutic response, and are promising biomarkers to predict the efficacy of immunotherapy [41, 42]. In the present study, two novel observations have been made. First, we used differentially expressed LMGs, including CYP19A1, FABP4, LRP2, SLCO1A2, PPARGC1A and ALOXE3, to construct the LMrisk for predicting prognosis of colon cancer, and found that the LMrisk was associated with the immunosuppressive TME and predictive biomarkers of immunotherapeutic response in colon cancer. To our knowledge, this is the first study that directly demonstrated that a prognostic risk model based on LMGs may predict immunotherapeutic response in colon cancer. Second, we uncovered for the first time that CYP19A1 inhibition downregulated PD-L1, IL-6 and TGF-β expression in colon cancer cells, and thereby enhanced the tumor-killing ability of CD8^+^ T cells. These results implicate the LMrisk based on the prognostic model as a predictive biomarker of immunotherapeutic response in colon cancer, highlighting its therapeutic potential for optimizing anti-PD-1 immunotherapy.

ALOXE3, PPARGC1A or FABP4 are prognostic biomarkers in colon cancer [43–45]. However, a single gene is difficult to provide powerful predictive performance for colon cancer patients [46]. Therefore, it is a trend to use a multiple gene model to predict prognosis of cancer patients [47]. Indeed, several studies have used multiple LMGs to construct prognostic models in patients with breast cancer, gastric cancer or osteosarcoma [48–50]. Here we integrated CYP19A1, FABP4, LRP2, SLCO1A2, PPARGC1A and ALOXE3 with clinicopathological information of patients to construct a prognostic nomogram in colon cancer. A recent study shows an 8-gene signature based on LMGs including RTN2, FYN, HEYL, FAM69A, FBXL5, HMGN2, LGALS4, STOX1 as a novel marker to predict colon cancer patients’ survival [8]. We reason that this discrepancy in prognosis-related LMGs could be due to different algorithms (differentially expressed LMGs *vs.* all LMGs). Our lipid metabolism-related nomogram has larger C-index than the model of Jiang et al. [8], suggesting its ability to better predict colon cancer prognosis as compared with gene signature-derived risk score. A previous study shows that nomogram-derived prognosis, together with user-friendly digital interfaces, elevates the accuracy of cancer survival prediction, and thereby allows for seamless incorporation to aid clinical decision making [47].

High level of MSI is closely related to the improved prognosis of colon cancer patients receiving immunotherapy [29], and higher TMB results in more neo-antigens, increasing chances for T cell recognition, accompanied by better ICI outcomes [30]. Here we demonstrated that the LMrisk was positively correlated with TMB and MSI, suggesting that the patients with high LMrisk are more likely to benefit from immunotherapy. Further analysis revealed that TTN mutation was the major reason for the high TMB in the colon cancer patients with high LMrisk. Previous studies show that TTN mutation is related to high immunogenicity and inflammatory tumor immune microenvironment in lung adenocarcinoma, accompanied by favorable objective response and survival with ICI administration [51, 52]. Along with immunogenicity, inflammatory TME makes colon cancer patients amenable to respond to ICIs [53]. We also observed more infiltration of macrophages, monocytes, NK cells and CAFs, and higher PD-L1 expression in colon cancer tissues from the patients with high LMrisk. Our extensive functional studies demonstrated that CYP19A1 protein expression was positively correlated with PD-L1 expression and infiltration of macrophages, CAFs and endothelial cells in human colon cancer tissues. Given that strong correlations of the LMrisk with PD-L1 expression, TMB and MSI, we conclude that the LMrisk including CYP19A1 is a promising biomarker for predicting immunotherapeutic response to colon cancer.

Several studies showed that men have a higher incidence of colon cancer than women, suggesting that estrogen may play a protective role in the development of colon cancer [54]. However, recent epidemiological studies have shown that hormone replacement therapy in postmenopausal women does not inhibit the development of colon cancer [55, 56]. Moreover, anti-PD-1 or anti-PD-L1 treatment resulted in a trend to greater overall survival and better response rates in individual males with colon cancer as compared with females [57]. Aromatase encoded by CYP19A1 and GPR30 are highly expressed in colon cancer tissues, and high expression of GPR30 predicts poor prognosis in colon cancer patients [58, 59]. Here, we demonstrated that CYP19A1 inhibition by letrozole or siRNA reduced production of IL-6 and TGF-β and downregulated PD-L1 expression by inactivating GPR30-Akt signaling, and thereby promoted the proliferation and cytotoxic activity of CD8^+^ T cells (Fig. S6). These data delineate that CYP19A1 inhibition combined with PD-1 antibody represents a promising therapeutic strategy for colon cancer.

According to our analyses, the LMrisk may predict response and outcome of immunotherapy in colon cancer. However, there are still some limitations that should be acknowledged. Firstly, because the mRNA expression data from colon cancer patients with immunotherapy is not available, the predictive ability of LMrisk for immunotherapeutic response is estimated indirectly by urothelial cancer cohorts and biomarkers. Therefore, further well-powered prospective studies are still needed. Secondly, we studied the expression and pathophysiological significance of CYP19A1 in vitro and in vivo, and further studies are needed on other LMGs including FABP4, LRP2, SLCO1A2, PPARGC1A and ALOXE3 in colon cancer.

## Conclusions

Collectively, we screened the six LMGs including CYP19A1, FABP4, LRP2, SLCO1A2, PPARGC1A and ALOXE3, and constructed a signature to predict prognosis and immunotherapeutic response in colon cancer, which was extendedly and externally validated. Importantly, CYP19A1 inhibition improves anti-PD-1 immunotherapy for colon cancer and blunts PD-L1-induced anergy of CD8^+^ T cells. Our findings facilitate prediction for prognosis and immunotherapeutic response in colon cancer, and targeting lipid metabolism reprogramming in the TME may be promising strategies for synergy with anti-PD-1 treatment.

## Supplementary Information

**Additional file 1: Fig. S1.** Stratified analysis of the LMrisk based on clinicopathological features including age, gender, T stage, N stage, M stage and TNM stage in TCGA dataset. **Fig. S2.** The LMrisk is an independent prognostic indicator in colon cancer. **Fig. S3.** Establishment and validation of the prognostic nomogram for colon cancer patients. **Fig. S4.** High CYP19A1 expression predicts poor prognosis and positively correlated with PD-L1 expression in the GEPIA webserver. **Fig. S5.** CYP19A1 inhibitor letrozole facilitates anti-PD-1 therapy in mice bearing orthotopic MC38 colon tumor. **Fig. S6.** A proposed mechanism to explain the role of CYP19A1 in tumor immune microenvironment in colon cancer. Supplementary Materials and Methods. **Supplementary Table S1.** The relationships between CYP19A1 expression and clinicopathological features including age, gender, T stage, N stage M stage and TNM stage in the tissue microarray. **Supplementary Table S2.** Univariate and multivariate Cox regression analyses of CYP19A1 expression in the human colon cancer tissue microarray. **Supplementary Table S3.** Effects of letrozole on body weight, biochemical profile and complete blood counts in the orthotopic MC38 tumor model.

## Data Availability

The datasets generated or analysed during the current study are available on reasonable request.

## Abbreviations

AA: Arachidonic acid
AUC: Areas under curve
CMS: Consensus molecular subtypes
COX: Cyclooxygenase
CYP: Cytochrome P450
GEO: Gene Expression Omnibus
GO: Gene Ontology
ICI: Immune checkpoint inhibitor
IHC: Immunohistochemistry
KEGG: Kyoto Encyclopedia of Genes and Genomes
LASSO: Least absolute shrinkage and selection operator
LMG: Lipid metabolism-related gene
LMrisk: Lipid metabolism-related gene prognostic risk score
MSI: Microsatellite instability
PBMCs: Peripheral blood mononuclear cells
PCA: Principal component analysis
PD-1: Programmed cell death protein 1
ROC: Receiver operating characteristic
TCGA: The Cancer Genome Atlas
TMB: Tumor mutational burden
TME: Tumor microenvironment
