# Supplementary material for: Targeting inhibition of prognosis-related lipid metabolism genes including CYP19A1 enhances immunotherapeutic response in colon cancer
Source: J Exp Clin Cancer Res. 2023 Apr 13;42:85. doi: 10.1186/s13046-023-02647-8 (PMC10100168; doi:10.1186/s13046-023-02647-8)
Supplement: Supplementary file 1 — Additional file 1: Fig. S1. Stratified analysis of the LMrisk based on clinicopathological features including age, gender, T stage, N stage, M stage and TNM stage in TCGA dataset. Fig. S2. The LMrisk is an independent prognostic indicator in colon cancer. Fig. S3. Establishment and validation of the prognostic nomogram for colon cancer patients. Fig. S4. High CYP19A1 expression predicts poor prognosis and positively correlated with PD-L1 expression in the GEPIA webserver. Fig. S5. CYP19A1 inhibitor letrozole facilitates anti-PD-1 therapy in mice bearing orthotopic MC38 colon tumor. Fig. S6. A proposed mechanism to explain the role of CYP19A1 in tumor immune microenvironment in colon cancer. Supplementary Materials and Methods. Supplementary Table S1. The relationships between CYP19A1 expression and clinicopathological features including age, gender, T stage, N stage M stage and TNM stage in the tissue microarray. Supplementary Table S2. Univariate and multivariate Cox regression analyses of CYP19A1 expression in the human colon cancer tissue microarray. Supplementary Table S3. Effects of letrozole on body weight, biochemical profile and complete blood counts in the orthotopic MC38 tumor model. [file 13046_2023_2647_MOESM1_ESM.zip › 13046_2023_2647_MOESM1_ESM/Supplementary material 23.02.28.docx]

**SUPPLEMENTARY MATERIAL**

**Targeting inhibition of prognosis-related lipid metabolism genes including CYP19A1 enhances immunotherapeutic response in colon cancer**

Lilong Liu^a #^, Min Mo^a #^, Xuehan Chen^a^, Dongchen Chao^a^, Yufan Zhang^a^, Xuewei Chen^b^, Yang Wang^a^, Nan Zhang^a^, Nan He^a^, Xi Yuan^a^, Honglei Chen^c, *^, Jing Yang^a, *^

**Supplementary Legends**

**Fig. S1 Stratified analysis of the LMrisk based on clinicopathological features including age, gender, T stage, N stage, M stage and TNM stage in TCGA dataset.** Kaplan–Meier survival curves showed the overall survival of high and low LMrisk colon cancer patients stratified by age (<66 years vs ≥66 years) **(A)**, gender (female vs male) **(B)**, TNM stage (stage I–II vs stage III–IV) **(C)**, N stage (N0 vs N1–N2) **(D)**, M stage (M0 vs M1) **(E)** and T stage (T1–T2 vs T3–T4) **(F)**, respectively.

**Fig. S2 The LMrisk is an independent prognostic indicator in colon cancer. (A, B)** Forest plot of univariate and multiple Cox regression analyses. **(C)** Multi-index receiver operating characteristic (ROC) curves of the LMrisk and clinical characteristics. (**D)** Heatmap of 6-lipid metabolism-related genes expression profiles and clinical characteristics of patients in high LMrisk and low LMrisk groups. (**E)** Associations between the LMrisk with tumor pathological features including TNM stage, T stage, N stage and M stage.

**Fig. S3 Establishment and validation of the prognostic nomogram for colon cancer patients. (A)** The nomogram was developed based on the LMrisk, age and TNM stage. (**B, C)** Calibration curves of the nomogram on 3- and 5-year survival probability. (**D, E)** Receiver operating characteristic (ROC) curves for 3- and 5-year overall survival prediction of the nomogram were performed in TCGA colon cancer dataset.

**Fig. S4 High CYP19A1 expression predicts poor prognosis and positively correlated with PD-L1 expression in the GEPIA webserver.** **(A)** CYP19A1 expression was significantly higher in tumor tissues compared with adjacent normal tissues in TCGA colon cancer dataset. **(B)** High CYP19A1 expression predicted poor prognosis in TCGA colon cancer dataset. **(C)** CYP19A1 gene expression was positively correlated with PD-L1 expression in TCGA colon cancer dataset.

**Fig. S5 CYP19A1 inhibitor letrozole facilitates anti-PD-1 therapy in mice bearing orthotopic MC38 colon tumor. A** Representative *ex vivo* images of colon with an orthotopic tumor in the cecum. **B** Tumor weights were measured in each group. **C** PD-L1 protein on tumor cells was determined by flow cytometry. **D** Representative immunohistochemistry (IHC) staining of CD8 and double staining for CD8 (red) and IFNγ (green) or GzmB (green) in the tumor tissues and their corresponding quantification analyses. Scale bar, 20 μm. **E** Intratumoral estradiol was detected by ELISA. **F** Hypoxia-inducible factor (HIF) -1α mRNA in the tumor tissues was detected by qPCR. **G** Representative immunofluorescence staining and frequency of CD31 (red) and αSMA (green) in the tumor tissues. Scale bar, 50 μm. The values are presented as the mean ± standard error of the mean, *n* = 6. ^*^, *P* < 0.05; ^**^, *P* < 0.01 *vs*. control. ^##^, *P* < 0.01 *vs*. α-PD-1.

**Fig. S6 CYP19A1 inhibition reduces productions of IL-6 and TGF-β and downregulates PD-L1 expression by inactivating GPR30-Akt signaling, and thereby promotes the proliferation and cytotoxic activity of CD8^+^ T cells.**

**Supplementary Materials and Methods**

**Mice**

C57BL/6 (male, 6–8 weeks old) and BALB/c mice (male, 6–8 weeks old) were purchased from the Centers for Disease Control and Prevention (Hubei, China). The mice were acclimatized for 1 week to adapt to the new environment before the experiment. The animals were housed in a pathogen-free environment on a 12 h light/dark cycle with ad libitum access to water and food. All animal experiments were performed in accordance with the policies of the animal ethics committee of the Animal Research Committee of Wuhan University, and maintained in accordance with the guidelines of the Association for Assessment and Accreditation of Laboratory Animal Care International.

**Cell culture**

Human colon cancer HT29 and HCT116 cell lines were purchased from the American Type Culture Collection (ATCC, Manassas, VA, USA). The HT29 and HCT116 cells were grown in DMEM containing 10% fetal bovine serum (FBS) in a humidified atmosphere of 5% CO_2_ and 95% air at 37°C. For all cell lines, mycoplasma testing using the MycoAlert Mycoplasma Detection kit (Lonza, Slough, UK) was performed every 3 months. Cells were regularly assessed based on their morphology before use.

**Cell proliferation assays**

The HT29 and HCT116 cells (1 × 10^4^/well) seeded in 96-well plates were treated with the indicated concentration of letrozole for 24 h. Cell viability was measured according to the protocol of 3-(4, 5-dimethylthiazol-2yl)-2,5-diphenyl tetrazolium bromide (MTT) (G111, Promega, WI, USA).

**Aromatase activity** **assays**

Aromatase activity was evaluated as previous described [1]. Briefly, HT29 and HCT116 cells were incubated with testosterone after the treatment with letrozole (5 μM), CYP19A1 siRNA or vehicle for 24 h. Culture media were collected at the indicated times, and processed to determine estradiol concentration by using an estradiol enzyme-linked immunosorbent assay (ELISA) kit (PE223, Beyotime, China) according to the manufacturer’s instructions. Cells were collected and protein was determined by Bradford assay.

**CYP19A1 siRNA transfection**

For CYP19A1 siRNA experiment, the HT29 and HCT116 cancer cells were transfected with 10 µM CYP19A1 siRNA (sc-41498, Santa Cruz, CA, USA). The negative control group was treated with the control siRNA in the same way.

**Isolation of PBMCs and their coculture with colon cancer cells**

Isolation of human peripheral blood mononuclear cells (PBMCs) from healthy volunteers were performed by density gradient centrifugation on Ficoll-Paque PREMIUM 1.077 g/ml as previously described [2]. All volunteers were healthy nonsmoking males with written informed consent. This experiment was approved by the Ethical Committee of the Medical School of Wuhan University. The *in vitro* coculture experiment of PBMCs with cancer cell was performed as previously described [3]. Briefly, the human PBMCs were incubated with target cells (human HT29 or HCT116 cells) at an effector to target (E : T) ratio of 5:1 for 24 h. The killing activity of PBMCs on human colon cancer cells was determined using flow cytometry.

**Flow cytometry analyses for tumor cell death and CD8^+^ T cell function**

Single-cell suspension from the coculture system treated with letrozole (5 μM), CYP19A1 siRNA or vehicle (DMSO or control siRNA) was labeled with EpCAM and 7-AAD or immunostained with PE-conjugated anti-CD3 antibody, APC-conjugated anti-CD8 antibody, PE-Cy7-conjugated anti-IFN-γ antibody and FITC-conjugated anti-CD107a antibody. Dead cells were excluded with Zombie NIR staining. FACS data were acquired using a CytoFLEX S flow cytometer (Beckman Coulter, Brea, CA, USA), and then analyzed using FlowJo software (Tree Star, Ashland, OR, USA). Results were expressed as the percentage of positive cells over the total number of cells.

**Western blot analyses**

Protein extracted from HT29 and HCT116 treated with the letrozole and CYP19A1 siRNA were resolved by SDS-PAGE and transferred to polyvinylidene fluoride membranes. The membrane was blocked with 5% nonfat milk and probed with primary antibodies against CYP19A1 (1:300, Santa Cruz, sc-374176), PD-L1 (1:1000, Abcam, ab213480), GPR30 (1:1000, Abcam, ab260033), Akt (1:1000, Abcam, ab8805), p-Akt (1:1000, Abcam, ab38449), HIF-α (1:1000, Abcam, ab179483) or GAPDH (1:5000, Abcam, ab8245). The antibodies were detected using peroxidase-conjugated anti-rabbit and anti-mouse immunoglobulin G (CST, Boston, USA), and blots were detected using the ECL system (Thermo Fisher Scientific, Waltham, MA, USA). The relative expression was normalized to the expression of GAPDH. Band intensity was quantified by densitometric analysis using the NIH ImageJ Program.

**Real-time quantitative PCR**

Total cellular RNA from was isolated with Trizol reagent (Invitrogen, Carlsbad, CA, United States) and reverse-transcribed into cDNA using a PrimeScript RT reagent kit (Takara Bio, Japan). The resulting cDNA was subsequently used as a template for the amplification of target gene transcripts by real-time PCR using SYBR Green PCR Master Mix (Takara Bio, Japan) on a CFX96 Real Time System (Bio–Rad, Hercules, CA). Relative expression levels of the target genes were normalized with the β-actin. Relative quantification was performed using the 2^−△△Ct^ method. Forward (F) and reverse (R) primers used were as follows: Human-TGF-β-F 5′-CCC ACA ACG AAA TCT ATG ACA AG-3′, Human-TGF-β-R 5′-CTA AGG CGA AAG CCC TCA AT -3′; Human-IL-6-F 5′- GGC ACT GGC CAG AAA ACA ACC-3′, Human-IL-6-R 5′-GCA AGT CTC CTC ATT GAA TCC-3′; Human-GzmB-F 5′-TGA AGC CAG GGC AGA CAT GC-3′, Human-GzmB-R 5′-GCC TCC AGA GTC CCC CTT AA-3′; Human-IFN-γ-F 5′-TGC AGG ACC CAT ATG TAA AAG A-3′, Human-IFN-γ-R 5′-TCA AAA TGC CTA AGA AAA G-3′; Human-β-actin-F 5′-AAG ATC ATT GCT CCT CCT GA-3′, Human-β-actin-R 5′-CTC GTC ATA CTC CTG CTT GCT-3′; Mouse-HIF-1α-F 5′-ACC TTC ATC GGA AAC TCC AAA G-3′, Mouse-HIF-1α-R 5′-CTG TTA GGC TGG GAA AAG TTA GG-3′; Mouse-β-actin-F 5′-CAT CCG TAA AGA CCT CTA TGC CAA C-3′, Mouse-β-actin-R 5′-ATG GAG CCA CCG ATC CAC A-3′.

**Preparation of nanoparticle-encapsulated CYP19A1 siRNA**

Hyaluronic acid-modified chitosan nanoparticles loaded with CYP19A1 siRNA were prepared as previously described [4]. Briefly, CYP19A1 siRNA solution and sodium tripolyphosphate solution were mixed in advance, and then slowly added into the chitosan solution during ultrasonication at 4°C to obtain hyaluronic acid -modified chitosan nanoparticles loaded with CYP19A1 siRNA.

**Tumor models and therapeutic efficacy study**

For subcutaneous colon cancer model, MC38 (2×10^5^) or CT26 (2×10^5^) cells were subcutaneously injected into the right flank of C57BL/6 or BALB/c mice. Orthotopic colon cancer model was generated as previously described [5,6]. Briefly, MC38 tumor chunks were implanted into the cecum wall by absorbable surgical sutures. On day 7 after implantation, mice were treated with CYP19A1 siRNA (250 μg/kg/3 days for 7 times, i.v.) or letrozole (10 mg/kg/day, five times weekly for 2 weeks, i.p.), anti-PD-1 antibody (200 μg/mouse every 3 days, i.p.), and anti-PD-1 antibody plus CYP19A1 siRNA (si-Scram as negative control) or letrozole. The dose of CYP19A1 siRNA and letrozole was selected according to the preliminary experiments. Tumor perfusion was measured by laser Doppler analysis as previously described [7]. 24 h after the last injection, the mice were euthanized and the tumors were collected and analyzed.

**Immunohistochemistry and Immunofluorescence staining**

Paraffin-embedded tissues from tumor-bearing mice after different treatments were processed and stained following standard protocols. To assess the distribution of CD8^+^ T cells, the sections from tumor-bearing mice after different treatments were stained with primary antibody mouse anti-CD8 (1:50, Santa Cruz Biotechnology, USA). 3,3-diaminobenzidine (DAB) colorimetric reagent solution from Dako (Carpinteria, CA, USA) was used and followed by hematoxylin counterstaining. Positive staining was quantified by three independent pathologists in a blinded fashion using image pro plus 6.0. For immunofluorescence staining, specimens were labeled with antibodies against CD31 (1:200, Abcam, ab24590), α-SMA (1:200, Abcam, ab7817), CD8a (1:200, Santa Cruz, sc-1177), granzyme B (1:200, Abcam, ab4059) and IFN-γ (1:200, Abcam, ab218426) overnight at 4°C for colocalization detection. After washing, the sections were incubated with a mixture of Alexa Fluor 488- and Alexa Fluor 594-conjugated secondary antibodies for 1 h. Each section was observed using an Olympus BX51 microscope with CCD DP80 (Olympus, Tokyo, Japan).

**Analysis for serum biochemical parameters**

Complete blood counts including red blood corpuscles, white blood corpuscles, platelet, mean cell volume, and hematocrit levels in the peripheral blood from the mice in indicated groups were measured by Sysmex XE-2100 (TOA Medical Electronics, Kobe, Japan), and the blood urea nitrogen, plasma alanine aminotransferase, aspartate aminotransferase, creatine kinase-MB, and creatine levels were measured by Beckman LX20 (Beckman-Coulter, Brea, CA) according to a previous study [8].

**Statistical analysis**

Statistical analyses and visualization were conducted using R software (version 4.0.1) and GraphPad Prism (version 8.0.1). The normal distribution of the data was assessed by the Kolmogorov–Smirnov test, and the number of repeats (*n*) for each experiment was specified in the figure legends. For normally distributed data, data were presented as mean ± standard error of the mean (SEM). Student’s t-test was used for two-group comparison, and one-way ANOVA followed by the Student–Newman–Keul’s test was used for multiple group comparisons. For non-normally distributed data, the Kruskal–Wallis test was used to compare three or more groups. Kaplan–Meier survival curve analysis and log-rank test were used to analyze overall survival. The Spearman rank correlation was used to test associations between the LMrisk and immune-related scores. All statistical tests were two-sided and *P* < 0.05 was considered statistically significant.

**References**

1. Ohno K, Araki N, Yanase T, Nawata H, Iida M. A novel nonradioactive method for measuring aromatase activity using a human ovarian granulosa-like tumor cell line and an estrone ELISA. Toxicological sciences : an official journal of the Society of Toxicology. 2004;82:443–50.

2. Song Q, Wang X, Wu X, Kang TH, Qin H, Zhao D, et al. IL-22-dependent dysbiosis and mononuclear phagocyte depletion contribute to steroid-resistant gut graft-versus-host disease in mice. Nat Commun. 2021;12:805.

3. Yang Z, Feng J, Xiao L, Chen X, Yao Y, Li Y, et al. Tumor-Derived Peptidoglycan Recognition Protein 2 Predicts Survival and Antitumor Immune Responses in Hepatocellular Carcinoma. Hepatology. 2020;71:1626–42.

4. Zhang W, Xu W, Lan Y, He X, Liu K, Liang Y. Antitumor effect of hyaluronic-acid-modified chitosan nanoparticles loaded with siRNA for targeted therapy for non-small cell lung cancer. Int J Nanomedicine. 2019;14:5287–301.

5. Liu X, Jiang J, Chan R, Ji Y, Lu J, Liao Y-P, et al. Improved efficacy and reduced toxicity using a custom-designed irinotecan-delivering silicasome for orthotopic colon cancer. ACS Nano. 2019;13:38–53.

6. Doleschel D, Hoff S, Koletnik S, Rix A, Zopf D, Kiessling F, et al. Regorafenib enhances anti-PD1 immunotherapy efficacy in murine colorectal cancers and their combination prevents tumor regrowth. J Exp Clin Cancer Res. 2021;40:288.

7. Wang C, Li Y, Chen H, Zhang J, Zhang J, Qin T, et al. Inhibition of CYP4A by a novel flavonoid FLA-16 prolongs survival and normalizes tumor vasculature in glioma. Cancer Lett. 2017;402:131–41.

8. Tang H, Liu Y, Wang C, Zheng H, Chen Y, Liu W, et al. Inhibition of COX-2 and EGFR by Melafolone Improves Anti-PD-1 Therapy through Vascular Normalization and PD-L1 Downregulation in Lung Cancer. J Pharmacol Exp Ther. 2019;368:401–13.

**Supplementary Tables**

**Supplementary Table S1.** The relationships between CYP19A1 expression and clinicopathological features including age, gender, T stage, N stage M stage and TNM stage in the tissue microarray.

| **Clinicopathological features** | **Cases** | **CYP19A1 expression** | | **χ^2^** | ***P* value** |
| --- | --- | --- | --- | --- | --- |
|  |  | **Low (*n* = 46)** | **High (*n* = 44)** |  |  |
| Age |  |  |  |  |  |
| ≤ 70 years | 46 | 29 | 17 | **5.361** | **0.034** |
| > 70 years | 44 | 17 | 27 |  |  |
| Gender |  |  |  |  |  |
| Female | 43 | 21 | 22 | 0.170 | 0.833 |
| Male | 47 | 25 | 22 |  |  |
| T stage |  |  |  |  |  |
| T1-T2 | 10 | 8 | 2 | 3.757 | 0.091 |
| T3-T4 | 80 | 38 | 42 |  |  |
| N stage |  |  |  |  |  |
| N0 | 56 | 35 | 21 | **7.695** | **0.009** |
| N1-N2 | 34 | 11 | 23 |  |  |
| M stage |  |  |  |  |  |
| M0 | 88 | 46 | 42 | 2.138 | 0.236 |
| M1 | 2 | 0 | 2 |  |  |

Abbreviations: CYP, Cytochrome P450; TNM, tumor-node-metastasis.

**Supplementary Table S2.** Univariate and multivariate Cox regression analyses of CYP19A1 expression in the human colon cancer tissue microarray.

| **Characteristics** | **Univariate analysis** | |  | **Multivariate analysis** | |
| --- | --- | --- | --- | --- | --- |
|  | **Hazard ratio**  **(95% CI)** | ***P* value** |  | **Hazard ratio**  **(95% CI)** | ***P* value** |
| Age (≤ 70 years *vs* >70 years) | 1.981 (1.114-3.521) | **0.020** |  | 1.579 (0.842-2.959) | 0.154 |
| Gender (Female *vs* Male) | 1.267 (0.719-2.233) | 0.413 |  | 1.763 (0.926-3.356) | 0.084 |
| T stage (T1-T2 *vs* T3-T4) | 1.269 (0.502-3.204) | 0.615 |  | 1.096 (0.332-3.623) | 0.881 |
| N stage (N0 *vs* N1) | 2.676 (1.519-4.714) | **0.001** |  | 2.931 (1.532-5.608) | **0.001** |
| M stage (M0 *vs* M1) | 11.016 (2.497-48.605) | **0.002** |  | 3.111 (0.462-20.940) | 0.243 |
| CYP19A1 expression (Low *vs* High) | 3.202 (1.754-5.846) | **<0.001** |  | 2.323 (1.192-4.525) | **0.013** |

Abbreviations: CI, confidence interval; TNM, tumor-node-metastasis.

**Supplementary Table S3.** Effects of letrozole on body weight, biochemical profile and complete blood counts in the orthotopic MC38 tumor model.

| Groups | Control | α-PD-1 | Letrozole | α-PD-1+letrozole |
| --- | --- | --- | --- | --- |
| Body weight (g) | 26.1 ± 0.7 | 26.2 ± 1.0 | 26.3 ± 1.2 | 26.9 ± 1.0 |
| ALT (IU/l) | 36.3 ± 2.1 | 36.5 ± 1.1 | 36.6 ± 0.9 | 36.3 ± 1.5 |
| BUN (mg/dl) | 26.4 ± 1.4 | 26.8 ± 2.4 | 26.8 ± 2.6 | 28.8 ± 2.0 |
| CREA (mg/dl) | 0.6 ± 0.1 | 0.5 ± 0.2 | 0.5 ± 0.2 | 0.5 ± 0.2 |
| AST (IU/l) | 105.8 ± 8.0 | 102.1 ± 5.9 | 99.9 ± 3.1 | 105.0 ± 7.2 |
| CK-MB (IU/l) | 153.7 ± 15.1 | 147.9 ± 17.6 | 123.8 ± 11.4 | 109.6 ± 13.8 |
| RBC (× 10^12^/l) | 8.4 ± 0.1 | 8.4 ± 0.2 | 8.3 ± 0.2 | 8.4 ± 0.2 |
| WBC (× 10^9^/l) | 3.7 ± 0.1 | 3.6 ± 0.2 | 3.6 ± 0.2 | 3.7 ± 0.2 |
| HCT (%) | 45.0 ± 2.8 | 43.8 ± 2.8 | 44.1 ± 3.1 | 46.4 ± 2.2 |
| MCV (fl) | 43.7 ± 3.7 | 44.7 ± 3.6 | 43.8 ± 3.6 | 45.8 ± 1.8 |
| PLT (× 10^9^/l) | 715.8 ± 20.7 | 726.1 ± 25.6 | 697.9 ± 13.7 | 706.9 ± 19.7 |

Abbreviations: ALT, alanine aminotransferase; BUN, blood urea nitrogen; CREA, creatine; AST, aspartate aminotransferase; CK-MB, creatine kinase-MB; RBC, red blood corpuscles; WBC, white blood corpuscles; HCT, hematocrit; MCV, mean cell volume; PLT, platelet. The values are presented as the mean ± standard error of the mean, *n* = 6.
