# Supplementary figures and images for: Targeting inhibition of prognosis-related lipid metabolism genes including CYP19A1 enhances immunotherapeutic response in colon cancer
Source: J Exp Clin Cancer Res. 2023 Apr 13;42:85. doi: 10.1186/s13046-023-02647-8 (PMC10100168; doi:10.1186/s13046-023-02647-8)

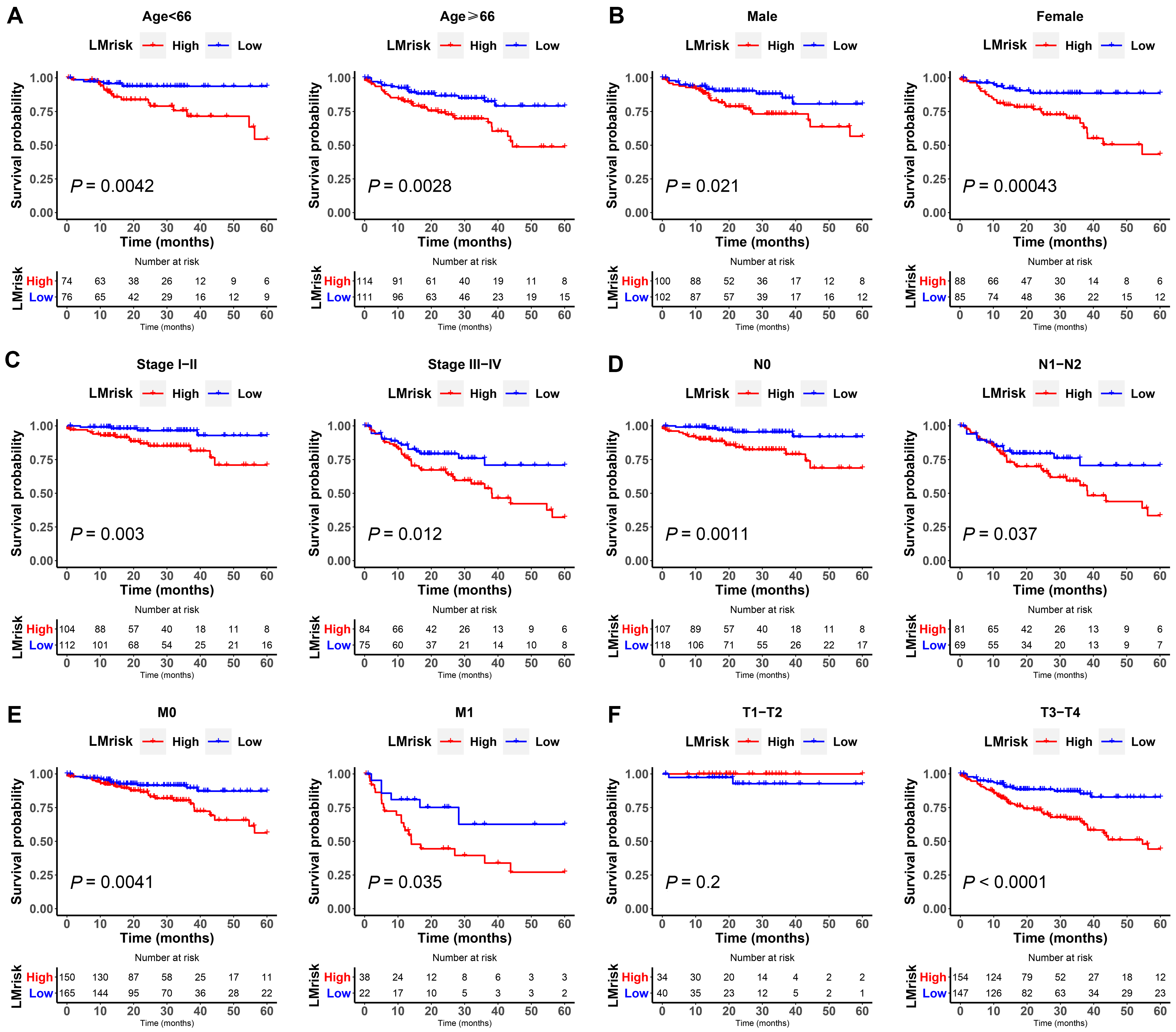

Supplement: Supplementary file 1 — Additional file 1: Fig. S1. Stratified analysis of the LMrisk based on clinicopathological features including age, gender, T stage, N stage, M stage and TNM stage in TCGA dataset. Fig. S2. The LMrisk is an independent prognostic indicator in colon cancer. Fig. S3. Establishment and validation of the prognostic nomogram for colon cancer patients. Fig. S4. High CYP19A1 expression predicts poor prognosis and positively correlated with PD-L1 expression in the GEPIA webserver. Fig. S5. CYP19A1 inhibitor letrozole facilitates anti-PD-1 therapy in mice bearing orthotopic MC38 colon tumor. Fig. S6. A proposed mechanism to explain the role of CYP19A1 in tumor immune microenvironment in colon cancer. Supplementary Materials and Methods. Supplementary Table S1. The relationships between CYP19A1 expression and clinicopathological features including age, gender, T stage, N stage M stage and TNM stage in the tissue microarray. Supplementary Table S2. Univariate and multivariate Cox regression analyses of CYP19A1 expression in the human colon cancer tissue microarray. Supplementary Table S3. Effects of letrozole on body weight, biochemical profile and complete blood counts in the orthotopic MC38 tumor model. [file 13046_2023_2647_MOESM1_ESM.zip › 13046_2023_2647_MOESM1_ESM/SFig 1.tif]

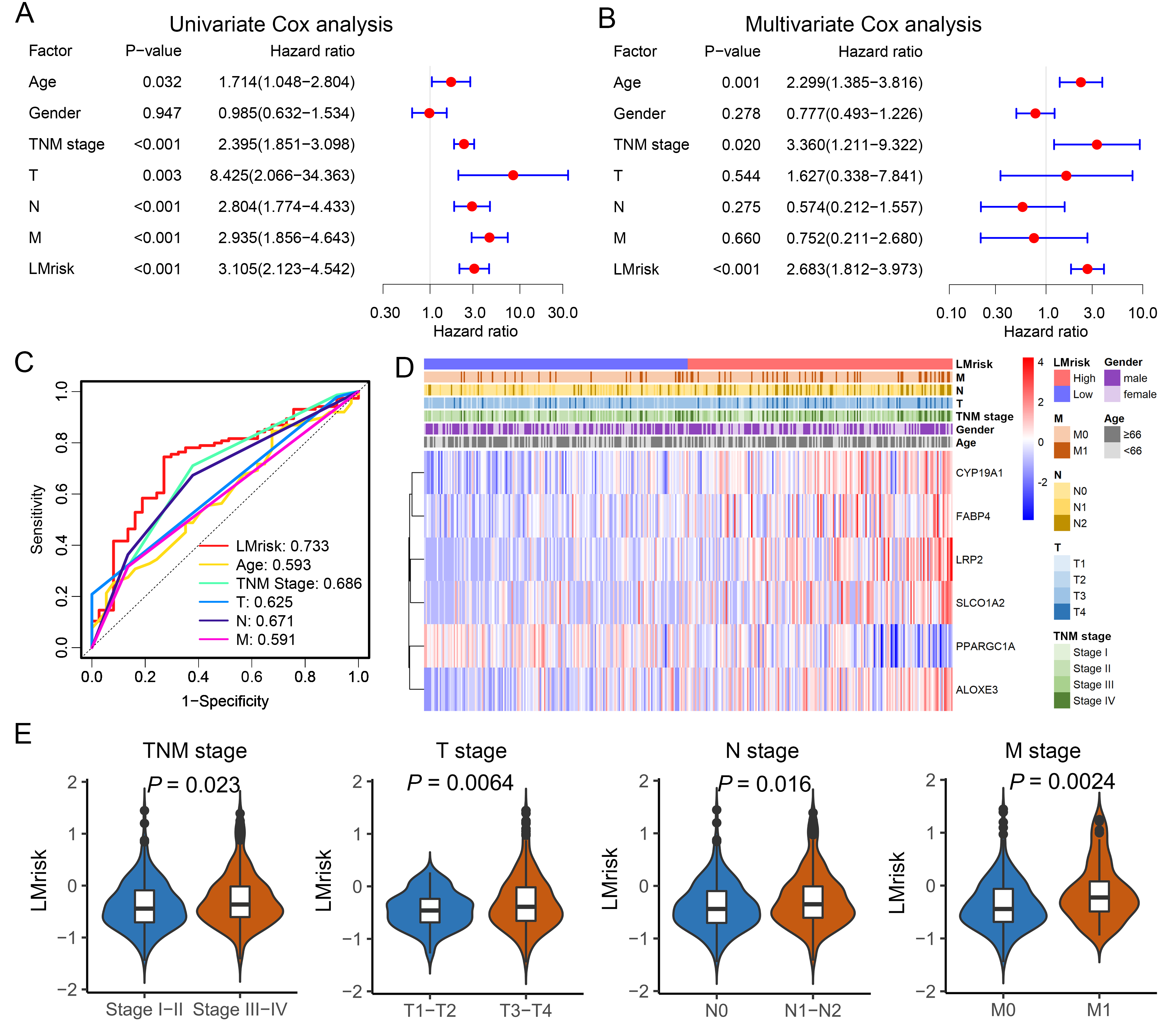

Supplement: Supplementary file 1 — Additional file 1: Fig. S1. Stratified analysis of the LMrisk based on clinicopathological features including age, gender, T stage, N stage, M stage and TNM stage in TCGA dataset. Fig. S2. The LMrisk is an independent prognostic indicator in colon cancer. Fig. S3. Establishment and validation of the prognostic nomogram for colon cancer patients. Fig. S4. High CYP19A1 expression predicts poor prognosis and positively correlated with PD-L1 expression in the GEPIA webserver. Fig. S5. CYP19A1 inhibitor letrozole facilitates anti-PD-1 therapy in mice bearing orthotopic MC38 colon tumor. Fig. S6. A proposed mechanism to explain the role of CYP19A1 in tumor immune microenvironment in colon cancer. Supplementary Materials and Methods. Supplementary Table S1. The relationships between CYP19A1 expression and clinicopathological features including age, gender, T stage, N stage M stage and TNM stage in the tissue microarray. Supplementary Table S2. Univariate and multivariate Cox regression analyses of CYP19A1 expression in the human colon cancer tissue microarray. Supplementary Table S3. Effects of letrozole on body weight, biochemical profile and complete blood counts in the orthotopic MC38 tumor model. [file 13046_2023_2647_MOESM1_ESM.zip › 13046_2023_2647_MOESM1_ESM/SFig 2.tif]

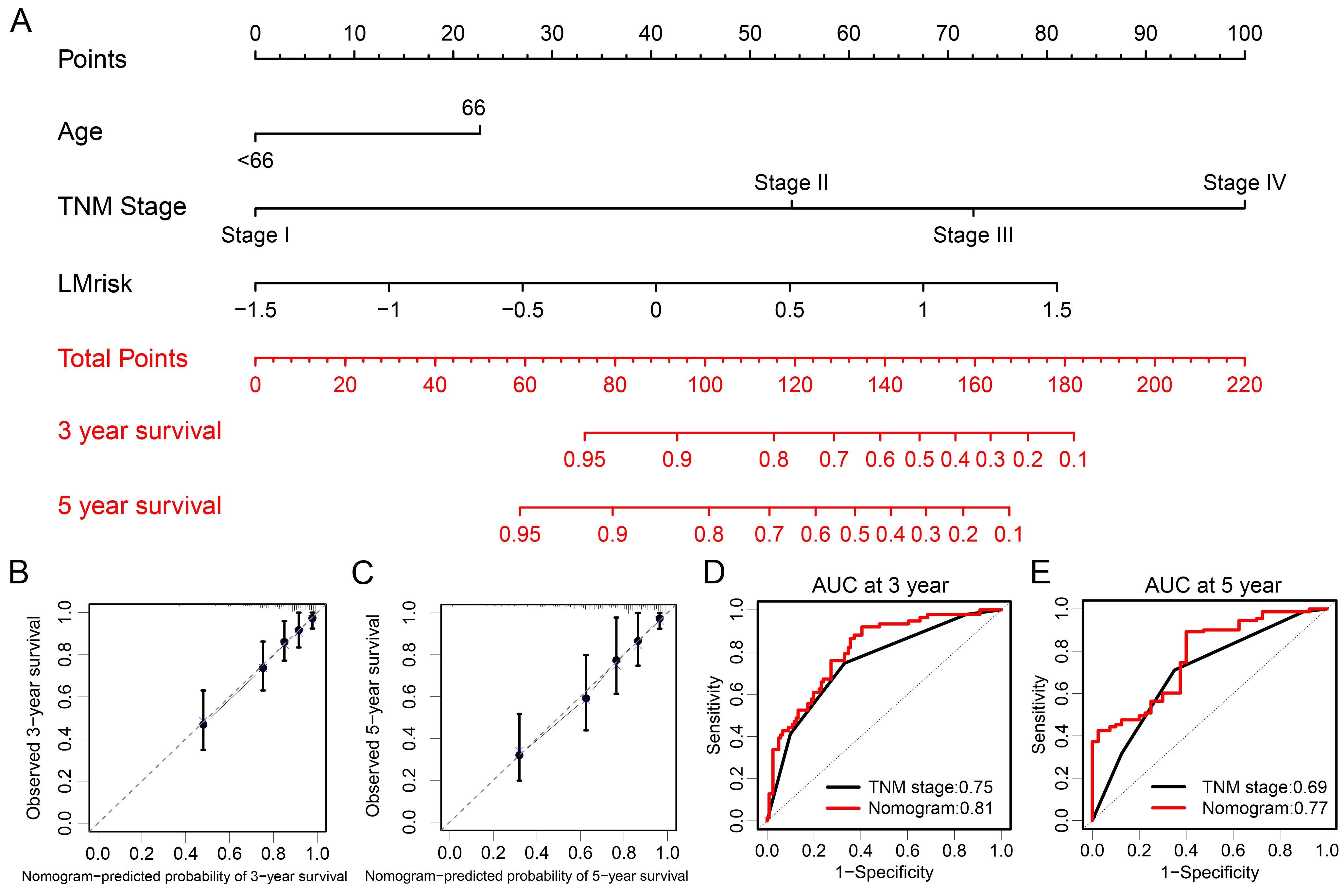

Supplement: Supplementary file 1 — Additional file 1: Fig. S1. Stratified analysis of the LMrisk based on clinicopathological features including age, gender, T stage, N stage, M stage and TNM stage in TCGA dataset. Fig. S2. The LMrisk is an independent prognostic indicator in colon cancer. Fig. S3. Establishment and validation of the prognostic nomogram for colon cancer patients. Fig. S4. High CYP19A1 expression predicts poor prognosis and positively correlated with PD-L1 expression in the GEPIA webserver. Fig. S5. CYP19A1 inhibitor letrozole facilitates anti-PD-1 therapy in mice bearing orthotopic MC38 colon tumor. Fig. S6. A proposed mechanism to explain the role of CYP19A1 in tumor immune microenvironment in colon cancer. Supplementary Materials and Methods. Supplementary Table S1. The relationships between CYP19A1 expression and clinicopathological features including age, gender, T stage, N stage M stage and TNM stage in the tissue microarray. Supplementary Table S2. Univariate and multivariate Cox regression analyses of CYP19A1 expression in the human colon cancer tissue microarray. Supplementary Table S3. Effects of letrozole on body weight, biochemical profile and complete blood counts in the orthotopic MC38 tumor model. [file 13046_2023_2647_MOESM1_ESM.zip › 13046_2023_2647_MOESM1_ESM/SFig 3.tif]

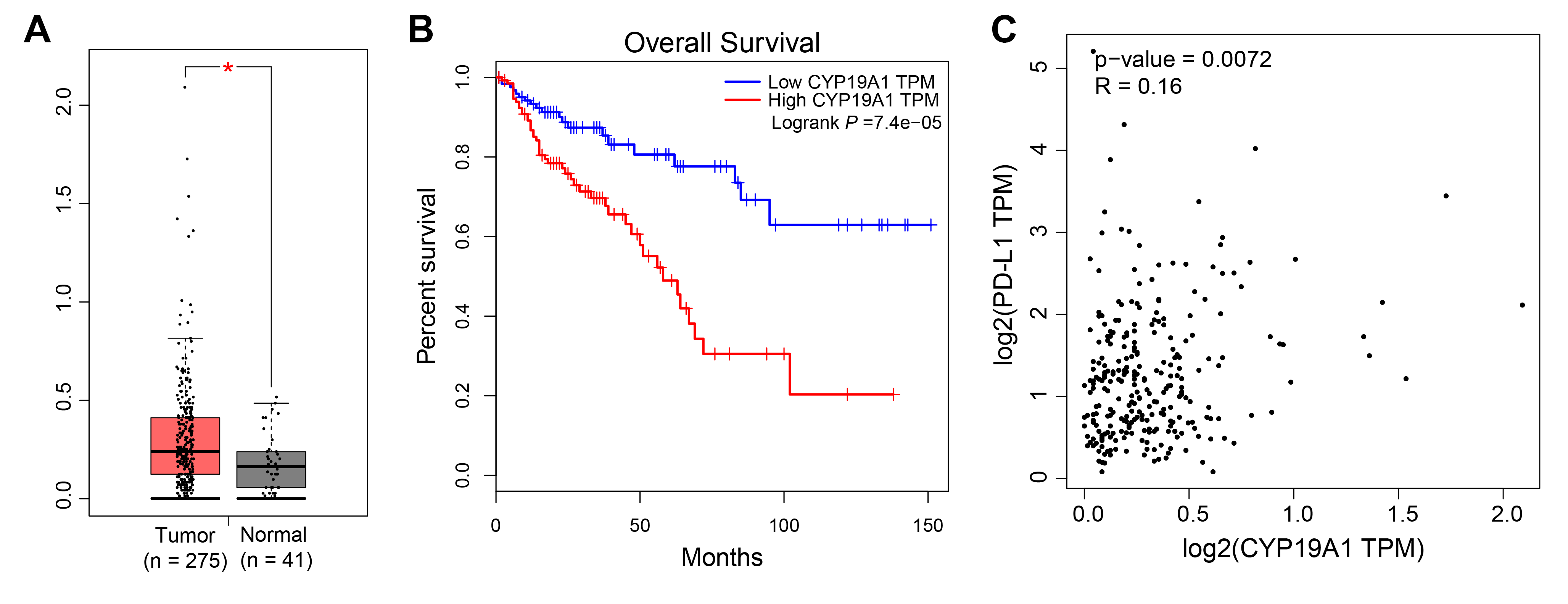

Supplement: Supplementary file 1 — Additional file 1: Fig. S1. Stratified analysis of the LMrisk based on clinicopathological features including age, gender, T stage, N stage, M stage and TNM stage in TCGA dataset. Fig. S2. The LMrisk is an independent prognostic indicator in colon cancer. Fig. S3. Establishment and validation of the prognostic nomogram for colon cancer patients. Fig. S4. High CYP19A1 expression predicts poor prognosis and positively correlated with PD-L1 expression in the GEPIA webserver. Fig. S5. CYP19A1 inhibitor letrozole facilitates anti-PD-1 therapy in mice bearing orthotopic MC38 colon tumor. Fig. S6. A proposed mechanism to explain the role of CYP19A1 in tumor immune microenvironment in colon cancer. Supplementary Materials and Methods. Supplementary Table S1. The relationships between CYP19A1 expression and clinicopathological features including age, gender, T stage, N stage M stage and TNM stage in the tissue microarray. Supplementary Table S2. Univariate and multivariate Cox regression analyses of CYP19A1 expression in the human colon cancer tissue microarray. Supplementary Table S3. Effects of letrozole on body weight, biochemical profile and complete blood counts in the orthotopic MC38 tumor model. [file 13046_2023_2647_MOESM1_ESM.zip › 13046_2023_2647_MOESM1_ESM/SFig 4.tif]

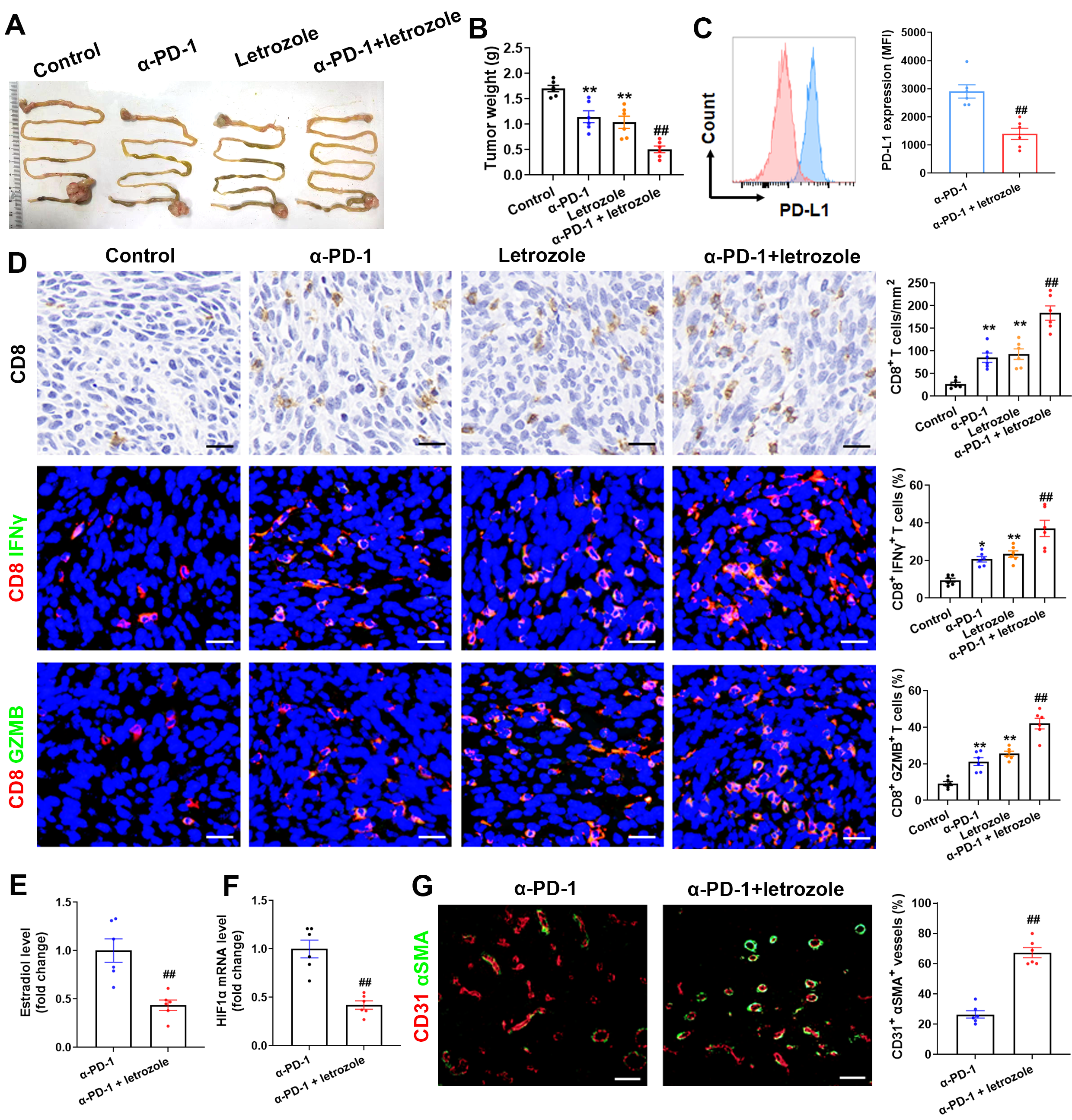

Supplement: Supplementary file 1 — Additional file 1: Fig. S1. Stratified analysis of the LMrisk based on clinicopathological features including age, gender, T stage, N stage, M stage and TNM stage in TCGA dataset. Fig. S2. The LMrisk is an independent prognostic indicator in colon cancer. Fig. S3. Establishment and validation of the prognostic nomogram for colon cancer patients. Fig. S4. High CYP19A1 expression predicts poor prognosis and positively correlated with PD-L1 expression in the GEPIA webserver. Fig. S5. CYP19A1 inhibitor letrozole facilitates anti-PD-1 therapy in mice bearing orthotopic MC38 colon tumor. Fig. S6. A proposed mechanism to explain the role of CYP19A1 in tumor immune microenvironment in colon cancer. Supplementary Materials and Methods. Supplementary Table S1. The relationships between CYP19A1 expression and clinicopathological features including age, gender, T stage, N stage M stage and TNM stage in the tissue microarray. Supplementary Table S2. Univariate and multivariate Cox regression analyses of CYP19A1 expression in the human colon cancer tissue microarray. Supplementary Table S3. Effects of letrozole on body weight, biochemical profile and complete blood counts in the orthotopic MC38 tumor model. [file 13046_2023_2647_MOESM1_ESM.zip › 13046_2023_2647_MOESM1_ESM/SFig 5.tif]

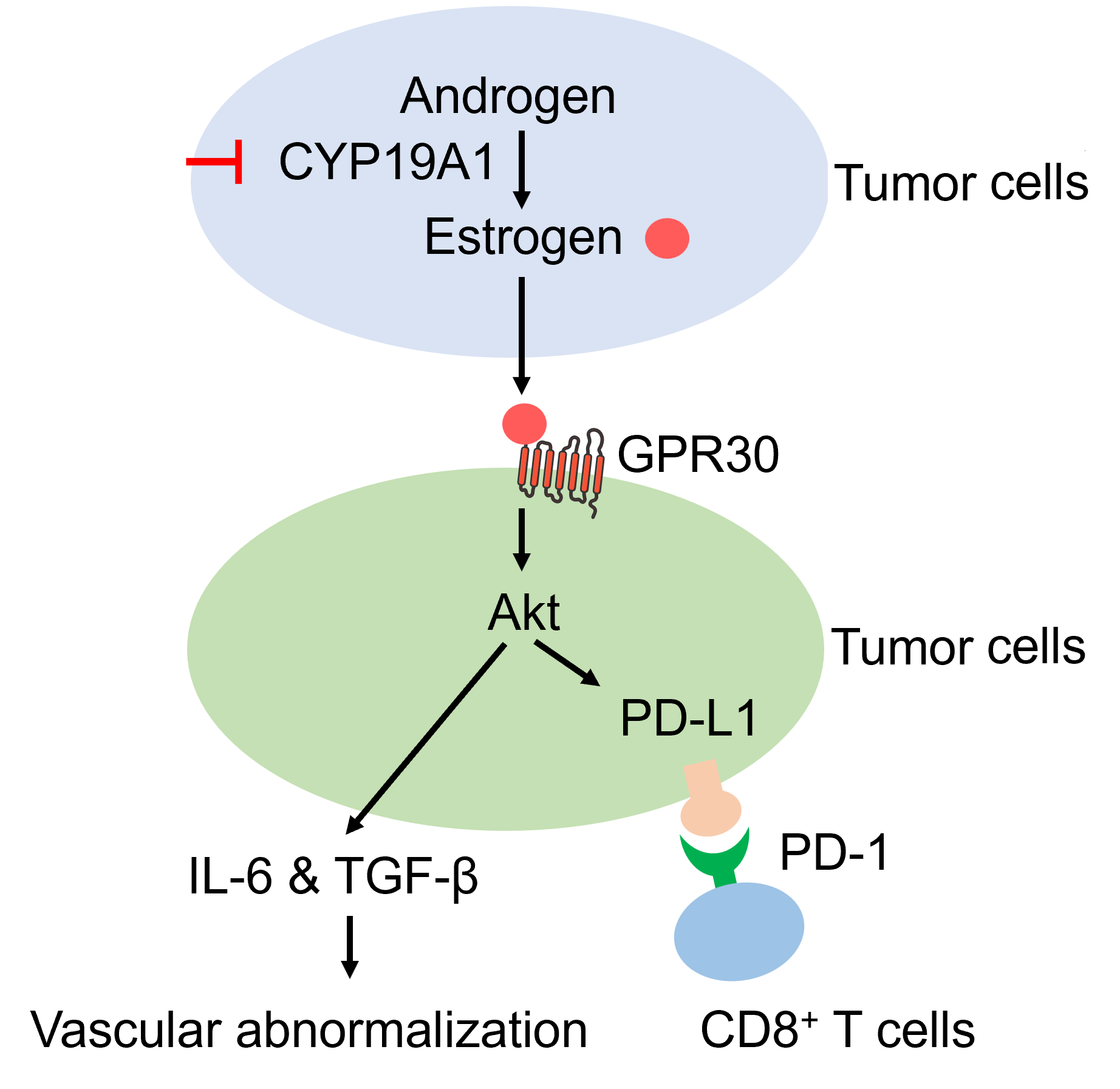

Supplement: Supplementary file 1 — Additional file 1: Fig. S1. Stratified analysis of the LMrisk based on clinicopathological features including age, gender, T stage, N stage, M stage and TNM stage in TCGA dataset. Fig. S2. The LMrisk is an independent prognostic indicator in colon cancer. Fig. S3. Establishment and validation of the prognostic nomogram for colon cancer patients. Fig. S4. High CYP19A1 expression predicts poor prognosis and positively correlated with PD-L1 expression in the GEPIA webserver. Fig. S5. CYP19A1 inhibitor letrozole facilitates anti-PD-1 therapy in mice bearing orthotopic MC38 colon tumor. Fig. S6. A proposed mechanism to explain the role of CYP19A1 in tumor immune microenvironment in colon cancer. Supplementary Materials and Methods. Supplementary Table S1. The relationships between CYP19A1 expression and clinicopathological features including age, gender, T stage, N stage M stage and TNM stage in the tissue microarray. Supplementary Table S2. Univariate and multivariate Cox regression analyses of CYP19A1 expression in the human colon cancer tissue microarray. Supplementary Table S3. Effects of letrozole on body weight, biochemical profile and complete blood counts in the orthotopic MC38 tumor model. [file 13046_2023_2647_MOESM1_ESM.zip › 13046_2023_2647_MOESM1_ESM/SFig 6.tif]
